# Gut microbiota-mediated associations of green tea and catechin intakes with glucose metabolism in individuals without type 2 diabetes mellitus: a four-season observational study with mediation analysis

**DOI:** 10.1007/s00203-023-03522-y

**Published:** 2023-04-14

**Authors:** Aoi Ito, Yuji Matsui, Masao Takeshita, Mitsuhiro Katashima, Chiho Goto, Kiyonori Kuriki

**Affiliations:** 1 Laboratory of Public Health, Graduate School of Integrated Pharmaceutical and Nutritional Sciences, University of Shizuoka, 52-1 Yada, Suruga-Ku, Shizuoka, Japan; 2 R&D - Health & Wellness Products Research, Kao Corporation, Tokyo, Japan; 3 Department of Health and Nutrition, Nagoya Bunri University, Aichi, Japan; 4 Laboratory of Public Health, School of Food and Nutritional Sciences, University of Shizuoka, Shizuoka, Japan

**Keywords:** Type 2 diabetes mellitus, Green tea, Catechin, Gut microbiota, Mediation analysis, Season

## Abstract

This four-season observational study aimed to examine the mediating role of the gut microbiota in the associations between green tea and catechin intakes and glucose metabolism in individuals without type 2 diabetes mellitus (T2DM). In each of the 4 seasons, 85 individuals without T2DM (56 male [65.9%]; mean [standard deviation] age: 43.3 [9.4] years) provided blood samples, stool samples, 3-day weighed dietary records, and green tea samples. Catechin intake was estimated by analyzing the tea samples. Linear mixed-effects model analysis showed that green tea intake was negatively associated with fasting blood glucose and insulin levels, even after considering the seasonal variations. Of the gut microbial species associated with green tea intake, the mediation analysis revealed that *Phocaeicola vulgatus* mediated the association between green tea intake and fasting blood glucose levels. These findings indicate that green tea can improve glucose metabolism by decreasing the abundance of *P. vulgatus* that is associated with elevated blood glucose levels in individuals without T2DM.

**Supplementary Information:**

The online version contains supplementary material available at 10.1007/s00203-023-03522-y.

## Introduction

For individuals with and without type 2 diabetes mellitus (T2DM), maintenance of blood glucose levels is important for the treatment and prevention of T2DM, respectively (Dinneen et al. 1998; American Diabetes Association 2021). Insulin plays a crucial role in the regulation of blood glucose levels (Stumvoll et al. 2005), with insulin resistance leading to hyperglycemia and acting as a major risk factor for T2DM (Fujimoto 2000). Hyperglycemia and insulin resistance are improved by the intake of catechins (Igarashi et al. 2007; Yan et al. 2012), which are key bioactive components of many teas, including black tea, green tea, and oolong tea (Nur et al. 2021). Among the various types of tea, green tea contains the highest levels of catechins, particularly epigallocatechin-3-gallate (EGCG) (Nur et al. 2021).

Previous epidemiological studies have reported that the consumption of green tea or/and catechins was associated with a reduced risk of T2DM development (Iso et al. 2006; Rienks et al. 2018; Nie et al. 2021). The antidiabetic effects of green tea and catechins are mediated by several mechanisms, including improved insulin resistance by increasing the expression of glucose transporter 4 (Wu et al. 2004), inhibition of intestinal glucose uptake through the suppression of α-amylase or α-glucosidase activity (Hara and Honda 1990; Yang and Kong 2016), and protection from pancreatic β-cell destruction by inhibition of nuclear factor-кB activation (Han 2003). A previous meta-analysis of 17 randomized control trials (RCTs) demonstrated that green tea decreased the fasting blood glucose, hemoglobin A1c (HbA1c), and insulin levels (Liu et al. 2013); however, majority of the examined RCTs supplied green teas with large amounts of catechins for only short periods of time (median: 12 weeks) (Liu et al. 2013). Although habitual dietary intakes, including green tea consumption, vary seasonally (Taguchi et al. 2015a, 2019) and seasonal variations in the diet can affect the glycemic status in humans (Ishii et al. 2001; Gikas et al. 2009), no studies have examined the effects of green tea intake and the seasonal variations in the diet.

Recent animal experiments suggested that the antidiabetic effects of green tea and catechins are, at least partly, mediated by the gut microbiota (Li et al. 2020; Zhang et al. 2020; Wu et al. 2021; Dey et al. 2020; Park et al. 2020; Chen et al. 2019; Wang et al. 2018, 2022; Zhou et al. 2022). Owing to their chemical complexity, catechins are poorly absorbed in the small intestine (Warden et al. 2001); thus, high amounts of catechins reach the colon where they are degraded by specific gut bacteria (Gowd et al. 2019). The gut microbiota is capable of metabolizing high-molecular-weight catechins to produce biologically active metabolites (Santangelo et al. 2019; Li et al. 2021). Green tea and its catechins and/or metabolites may have important impacts on the gut microecology (Liu et al. 2020; Zhou et al. 2022). Green tea and catechins were reported to inhibit the growth of pathogenic bacteria, such as *Clostridium* spp. (Sakanaka et al. 2000; Cui et al. 2012; Zhang et al. 2013), and promote the growth of potentially beneficial bacteria, such as *Bifidobacterium* and *Lactobacillus* (Tzounis et al. 2008; Zhang et al. 2013; Sun et al. 2018). Moreover, previous animal experiments revealed that alterations in the gut microbiota induced by green tea or catechins intake were correlated with improvements in blood glucose levels (Chen et al. 2019; Wu et al. 2021) and insulin resistance (Dey et al. 2020). Similarly, in individuals without T2DM, intake of green tea and catechins modulated the composition of the gut microbiota (Okubo et al. 1992; Jin et al. 2012; Most et al. 2017; Yuan et al. 2018), although most of the studies only analyzed selected bacteria using culture-based methods (Okubo et al. 1992) or real-time polymerase chain reaction (Jin et al. 2012; Most et al. 2017). Furthermore, no studies have examined the mediating role of the gut microbiota in the associations of green tea and catechins intakes with glucose metabolism in humans.

Hence, the present study aimed (1) to evaluate whether daily intakes of green tea and catechins are associated with biomarkers of glucose metabolism and the relative abundance of gut microbiota in individuals without T2DM in the four seasons and (2) to examine whether the gut microbiota mediates the associations between daily intakes of green tea and catechins and biomarkers of glucose metabolism.

## Methods

### Study design and participants

In this 4-season observational study, 91 participants who lived in Shizuoka, Japan, were recruited from January 2013 to March 2014 as part of the Sakura Diet Study, a sub-study of the Japan Multi-Institutional Collaborative Cohort Study in the Shizuoka-Sakuragaoka area (Endoh et al. 2017; Akimoto et al. 2019). Blood samples, stool samples, 3-day weighed dietary records (WDRs), and green tea samples were collected from the participants in each of the four seasons (winter, spring, summer, and autumn, with winter set as the first season). None of the participants self-reported a history of T2DM or taking antidiabetic medications. (1) Individuals who were suspected of having T2DM (fasting blood glucose of ≥ 126 mg/dL and/or HbA1c of ≥ 6.5%) based on the American Diabetes Association criteria (American Diabetes Association 2021) (*n* = 3), (2) individuals who reported taking antibiotics in each of the four seasons (winter: *n* = 4; spring: *n* = 1; summer: *n* = 2; and autumn: *n* = 0), and (3) individuals who did not provide blood and stool samples in any season for reasons such as busy lifestyles (*n* = 3) were excluded.

The participants received a verbal explanation about the purpose of the study and signed an informed consent form prior to their participation in the study. The study was conducted in accordance with the principles of the Declaration of Helsinki and was approved by the Ethics Committee of The University of Shizuoka (no. 24-24). The results were reported according to the Strengthening the Reporting of Observational Studies in Epidemiology-Nutritional Epidemiology (Lachat et al. 2016).

### Dietary assessment and lifestyle factors

The daily intakes of energy, ethanol, and green tea were assessed in each of the four seasons using non-continuous 3-day WDRs (2 weekdays and 1 weekend, a total of 12 days), based on the 2015 Standard Tables of Food Composition in Japan (seventh revised edition) (Ministry of Education, Culture, Sports, Science and Technology, Japan 2015). All WDRs were systematically reviewed by two trained registered dieticians. The daily green tea intake was calculated based on the average value of the 3-day WDRs and adjusted for the total energy intake using the density method (Willett and Stampfer 1986). In addition to the 3-day WDRs, green tea samples were collected and analyzed to estimate the catechins and EGCG intakes without dietary supplementation (Endoh et al. 2017). Briefly, each time the participants consumed green tea, they were asked to collect samples of the tea in plastic bottles containing ascorbic acid. EGCG, epicatechin gallate, epigallocatechin, epicatechin, gallocatechin gallate, catechin gallate, gallocatechin, and catechin were individually analyzed in the tea samples using high-performance liquid chromatography with ultraviolet detection; the sum of the intakes of all eight catechins was defined as the catechins concentration. The inter-assay coefficients of variation for the concentrations of catechins and EGCG were 1.5% and 4.4%, respectively (Endoh et al. 2017). The daily catechins and EGCG intakes were estimated using formulas (1) and (2) shown below and adjusted for total energy intake using the density method (Willett and Stampfer 1986).

1 $${\text{Catechins}}\;{\text{intake}}\;{\text{(mg/day)}} = {\text{Catechins}}\;{\text{concentration}}\;{\text{(mg/mL)}} \times {\text{green}}\;{\text{tea}}\;{\text{intake}}\;{\text{(mL/day)}}$$

2 $${\text{EGCG}}\;{\text{intake}}\;{\text{(mg/day)}} = {\text{EGCG}}\;{\text{concentration}}\;{\text{(mg/mL)}} \times {\text{green}}\;{\text{tea}}\;{\text{intake}}\;{\text{(mL/day)}}$$

Information about the participants’ medical history and lifestyle factors, including smoking status (never smoker, past smoker, or current smoker) and physical activity, were collected via a self-administered lifestyle questionnaire at the beginning of the study (winter). Physical activity was estimated based on the metabolic equivalents (METs) of daily and leisure-time activity. The METs•h/day value was calculated by multiplying the reported daily time spent in each activity by the assigned MET intensity (Hara et al. 2012).

### Anthropometric and biochemical measurements

In each of the four seasons, height and weight were measured to the nearest 0.1 cm and 0.1 kg, respectively, and blood samples were collected after evaluating the antidiabetic drug use using the self-administered medical questionnaire. Body mass index (BMI) was calculated by dividing the weight in kilograms by height in meter squared (m^2^). The HbA1c, fasting blood glucose, and insulin levels were measured using an enzymatic method, ultraviolet absorption spectrophotometry, and chemiluminescent enzyme immunoassay, respectively, in the SRL Clinical Laboratory. The homeostatic model assessment index of insulin resistance (HOMA-IR) was calculated to estimate the degree of insulin resistance (Wallace et al. 2004): HOMA-IR = fasting blood insulin (μU/mL) × fasting blood glucose (mg/dL)/405.

### Stool sample collection, deoxyribonucleic acid extraction, and 16S ribosomal ribonucleic acid gene sequencing

The participants were requested to collect stool samples and complete the self-administered medical questionnaire on antibiotic use in each of the four seasons (Hisada et al. 2015). Briefly, each stool sample was suspended in 100 mM Tris–HCl (pH 9), 40 mM ethylenediaminetetraacetic acid, 4 M guanidine thiocyanate, and 0.001% bromothymol. The fecal solids in the suspension were broken down using a FastPrep-24 Instrument (MP Biomedicals, Santa Ana, CA, USA) with zirconia beads at 5 m/s for 2 min. Bacterial deoxyribonucleic acid (DNA) was extracted using a Magtration System 12GC (Precision System Science, Chiba, Japan). The bacterial community DNA was amplified for the V3–V4 region of 16S ribosomal ribonucleic acid (rRNA) genes (Takahashi et al. 2014), and sequencing was performed using a paired-end protocol modified to 2 × 300-bp cycles in an Illumina MiSeq sequencing system (Illumina, San Diego, CA, USA) and a MiSeq Reagent Kit version 3 (600 Cycles; Illumina). After demultiplexing, a clear overlap in the paired-end reads was observed. After quality filtering, reads with a quality value score of ≥ 20 for more than 99% of the sequence were extracted. Bacterial identification from the sequences was performed using the Metagenome@KIN analysis software (World Fusion, Tokyo, Japan) and the TechnoSuruga Lab Microbial Identification database DB-BA 9.0 (TechnoSuruga Laboratory, Shizuoka, Japan). Several of these species have been re-classified, as defined in the “List of Prokaryotic names with Standing in Nomenclature” database accessed on February 10 2023 (Parte et al. 2020).

Considering the measurement precision, gut microbiota with a relative abundance of > 0.1% were filtered (Hisada et al. 2015). To address the issue of zero-inflated data, gut microbial species detected in at least 50% of the samples were selected; hence, only 41 genera and 71 species were included for further analysis, which were subjected to a robust centered-log-ratio transformation to the relative abundance data using the “decostand” function in the “vegan” package (version 2.6-4) (Aitchison 1982). The relative abundance at the phylum level was calculated as the sum of the relative abundances at the genus level belonging to each phylum. To evaluate the alpha-diversity, Shannon–Wiener diversity indices were calculated using the “diversity” function in the “vegan” package (version 2.6-4) (Oksanen et al. 2022).

### Handling of missing values

The percentages of missing data from the self-administered questionnaires, blood samples, anthropometric measurements, stool samples, 3-day WDRs, and green tea samples were 1.2%, 11.9%, 12.5%, 14.5%, 16.6%, and 20.5%, respectively. Multiple imputations were used to create and analyze 50 multiple imputed datasets with 20 interactions. The missing values were imputed under fully conditional specification using the “miceadds” (version 3.15-21) and “mice” package (version 3.14-0) (van Buuren and Groothuis-Oudshoorn 2011). The parameters of substantive interest were estimated separately for each imputed dataset and combined using Rubin’s roles (Rubin 1987). Trace and density plots for each variable were constructed to monitor the convergence of multiple imputations. The imputations created in the study were based on the assumption that the data were missing at random (MAR); thus, sensitivity analyses using the delta-adjustment method were performed to confirm the plausibility of the MAR assumption (Rubin 1987; van Buuren and Groothuis-Oudshoorn 2011). The delta-values were set for five different situations with the green tea intakes ranging from − 200 to + 200 mL/1,000 kcal rather than that assumed in the MAR.

### Statistical analysis

Categorical variables were expressed as number and percentage, while continuous variables were expressed as mean and standard deviation (SD) or median and interquartile range (IQR) depending on the distribution determined by visualization in the relevant histogram. The seasonal variations in continuous variables were analyzed by repeated-measures analysis of variance (ANOVA) with Dunnett’s multiple comparisons using winter as the reference.

The associations between green tea and catechins intakes, the relative abundance of gut microbiota, and the biomarkers of glucose metabolism (fasting blood glucose, HbA1c, insulin, and HOMA-IR levels) were analyzed using a multiple regression model based on the data in the first season (i.e., winter) and a linear mixed-effects model with a random intercept based on the data in all four seasons using the “lme4” package (version 1.1-27), respectively. Both models were adjusted for age, sex (categorical variable; female as reference), smoking status (categorical variable; non-smoker as reference), BMI, energy intake, ethanol intake, and physical activity. The linear mixed-effects model was further adjusted for seasons (categorical variable; winter as reference) and included the identification of participants as a random intercept to account for repeated measurements within participants. These variables were selected based on the covariates used in previous studies investigating the associations between green tea intake and glucose metabolism or risk of T2DM (Iso et al. 2006; Jing et al. 2009). For multiple testing of the gut microbiota, the false discovery rate *q* values were calculated based on the observed *p* values for each taxonomical level using the “qvalue” function in the “qvalue” package (version 2.31.1) (Storey and Tibshirani 2003). If *p *value of < 0.05 and *q *value of < 0.20 were observed, the finding was considered statistically significant.

A model-based mediation analysis was also performed to examine the gut microbiota-mediated associations of green tea and catechins intakes with the biomarkers of glucose metabolism using the “mediate” function in the “mediation” package (version 4.5-0) (Tingley et al. 2014). Using this approach, the total effect of green tea and catechins intakes on the biomarkers of glucose metabolism was divided into the average direct effect (ADE) of exposure and the average causal mediation effect (ACME) accounting for a mediator (Imai et al. 2011). The gut microbial taxa associated with green tea or catechins intake was selected as a potential mediator in the mediation analysis. Two linear mixed-effects models were fitted with random intercept, with one modeling the exposure–mediator association and the other modelling the mediator–outcome association. The variables used for the outcomes and mediators were modeled as continuous variables. The exposure variables (i.e., green tea and catechins) were categorized into low- and high-intake groups based on their median values to make the contrast in the mediation analysis (Imai et al. 2010). The adjusted covariates were the same as those in the linear mixed-effects model. Point estimates and confidence intervals (CIs) were estimated using the quasi-Bayesian Monte Carlo approximation method with 1,000 re-samplings in the mediation analysis (Tingley et al. 2014). The proportion mediated was the proportion of the total effect due to a mediator, which was calculated as the mediation effect of the gut microbiota divided by the total effect of green tea or catechins intake on the biomarkers of glucose metabolism. Because the current version of the “mediation” package cannot perform a sensitivity analysis for multiple imputed and multilevel datasets (Tingley et al. 2014), mediation analysis of the imputed dataset was conducted without performing a sensitivity analysis. A two-sided value of *p* < 0.05 was considered statistically significant. All statistical analyses were performed using R software, version 4.1.1 (R Foundation for Statistical Computing, Vienna, Austria).

## Results

### Characteristics of the participants and gut microbiota

A total of 85 participants (65.9% male; mean age: 43.3 years) without T2DM were included. The participant characteristics are presented in Table 1. In the first season, the mean (SD) levels of fasting blood glucose, HbA1c, insulin, and HOMA-IR were 91.8 (8.9) mg/dL, 5.3 (0.3)%, 5.8 (4.5) µU/mL, and 1.3 (1.1), respectively. The mean (SD) intakes of total energy and green tea were 1975 (390) kcal and 443 (417) mL/day, respectively. The median (IQR) intakes of catechins and EGCG were 67.8 (23.2–150.1) and 9.5 (0.9–26.9) mg/day, respectively. Seasonal variations were observed in the daily intakes of green tea and EGCG (repeated-measures ANOVA* p* < 0.05), with green tea intake being significantly lower in spring, summer, and autumn than in winter (Dunnett’s test* p* < 0.05).

**Table 1:** Participants’ characteristics in all four seasons

		Season^a^								*p*^b^
		Winter (*n* = 81)		Spring (*n* = 84)		Summer (*n* = 83)		Autumn (*n* = 85)		
Sex, *n* (%)	Male	55	67.9	56	66.7	55	66.3	56	65.9	
	Female	26	32.1	28	33.3	28	33.7	29	34.1	
Smoking status, *n* (%)^c^	Never-smoker	53	65.4	56	66.7	55	66.3	56	65.9	
	Past-smoker	14	17.3	15	17.9	14	16.9	15	17.6	
	Smoker	14	17.3	13	15.5	14	16.9	14	16.5	
Age (years)^c^		43.4	9.4	43.4	9.4	43.4	9.4	43.3	9.4	
Physical activity (METs•h/day)^c^		10.6	6.8	10.7	7.0	10.8	6.9	10.7	6.9	
BMI (kg/m^2^)		23.3	3.8	23.0	3.8	22.9	3.6	23.0	3.4	0.18
Fasting blood glucose (mg/dL)		91.8	8.9	91.3	7.7	91.6	8.5	90.4	8.5	0.20
HbA1c (%)		5.3	0.3	5.3	0.2	5.3	0.2	5.3	0.2	0.23
Insulin (μU/mL)		5.8	4.5	5.6	4.3	5.3	4.6	6.5	9.2	0.55
HOMA-IR		1.3	1.1	1.3	1.1	1.2	1.2	1.5	2.2	0.66
Shannon diversity index^d^		2.72	0.27	2.61	0.23	2.62	0.22	2.6	0.22	** < 0.001**
Energy intake (kcal/day)		1975	390	1899	470	1906	442	1953	461	0.06
Ethanol intake (g/day)		0.6	0.2, 6.0	0.4	0.1, 9.9	0.5	0.2, 8.9	0.4	0.1, 4.5	0.25
Green tea (mL/day)^d^		443	417	341	365	333	333	372	358	** < 0.01**
Catechins (mg/day)		67.8	23.2, 150.1	61.2	1.1, 176.6	84.9	0.6, 184.9	90.7	25.4, 250.1	0.34
EGCG (mg/day)		9.5	0.9, 26.9	7.6	0.1, 27.6	8.3	0.1, 37.3	18.2	4.5, 49.8	**0.046**
Green tea (mL/1,000 kcal)^e^		229	226	186	195	179	180	200	196	**0.049**
Catechins (mg/1,000 kcal)		35.0	11.6, 75.6	29.4	0.8, 116.5	43.4	0.3, 127.9	42.4	11.2, 146.1	0.51
EGCG (mg/1,000 kcal)		5.3	0.5, 13.9	3.7	0.04, 14.6	3.8	0.1, 17.3	9.8	2.1, 29.5	0.09

METs, metabolic equivalents; BMI, body mass index; HbA1c, glycated hemoglobin A1c; HOMA-IR, homeostatic model assessment index of insulin resistance; EGCG, epigallocatechin-3-gallate

^a^Continuous data are expressed as mean and standard deviation or median and interquartile range, while categorical data are expressed as number and percentage

^b^For comparisons between the seasons, *p* values were estimated by repeated-measures analysis of variance, and *p *values of < 0.05 are shown in bold

^c^The following variables were measured only in the first season (winter), and their values were used as representative values for the remaining three seasons: age, smoking status, and physical activity

^d^Lower in spring, summer, and autumn compared with that in winter (Dunnett’s multiple comparisons test *p* < 0.05)

^e^Lower in summer compared with that in winter (Dunnett’s multiple comparisons test *p* < 0.05)

**Fig. 1:**
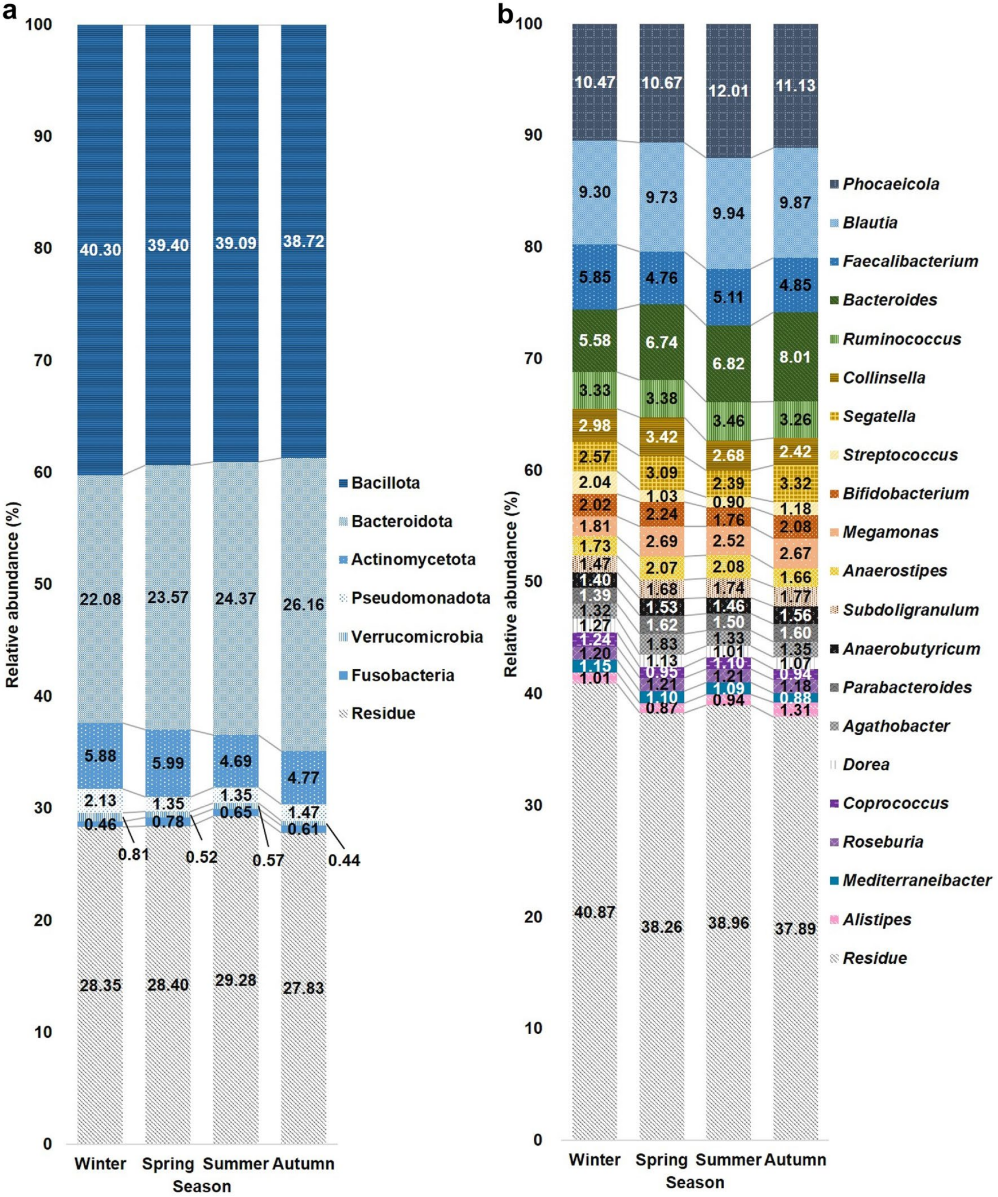
Characteristics of the human gut microbiota at the phylum (**a**) and genus levels (**b**) in each of the four seasons. Each bar chart shows the relative abundance of 6 most abundant phyla and 20 most abundant genera. Other bacterial phyla and genera were combined as residue

Among the gut bacteria analyzed, 6 phyla, 41 genera, and 71 species were selected for further analysis. The relative abundances of the gut microbiota at the phylum and genus levels in the four seasons are shown in Fig. 1. In the first season, the mean (SD) relative abundances of the three dominant phyla were 40.3% (8.0%) for Bacillota, 22.1% (8.6%) for Bacteroidota, and 5.9% (4.3%) for Actinomycetota (Fig. 1a); the mean (SD) relative abundances of the three dominant genera were 10.5% (7.0%) for *Phocaeicola*, 9.3% (4.9%) for *Blautia*, and 5.9% (3.7%) for *Faecalibacterium* (Fig. 1b)*.* The mean (SD) relative abundances of the five dominant species were 5.9% (3.7%) for *Faecalibacterium prausnitzii*, 4.7% (4.0%) for *Blautia wexlerae*, 3.1% (4.0%) for *Phocaeicola vulgatus* (formerly *Bacteroides vulgatus*), 2.9% (3.0%) for *Collinsella aerofaciens*, and 2.8% (4.6%) for *Phocaeicola dorei* (formerly *Bacteroides dorei*).

### Associations of green tea and catechin intakes with the biomarkers of glucose metabolism

Green tea intake was negatively associated with fasting blood glucose and insulin levels (fasting blood glucose: *β* =  − 4.89 × 10^−3^ [95% CI, − 9.64 × 10^−3^ to − 1.33 × 10^−4^], *p* = 0.04; insulin: *β* =  − 4.43 × 10^−4^ [95% CI, − 7.98 × 10^−4^ to − 8.82 × 10^−5^], *p* = 0.01; Table 2) in the four seasons, with fasting blood glucose levels being significantly lower in autumn than in winter. Catechins and EGCG intakes were marginally associated with lower HbA1c levels in the first season (catechins: *β* =  − 5.55 × 10^−4^ [95% CI, − 1.13 × 10^−3^ to 2.21 × 10^−5^], *p* = 0.06; EGCG: *β* =  − 1.65 × 10^−3^ [95% CI, − 3.39 × 10^−3^ to 8.06 × 10^−5^], *p* = 0.06; Table 2), but not in the four seasons (*p* > 0.05; Table 2). The sensitivity analyses revealed similar associations (Table S1).

**Table 2:** Associations of green tea and catechin intakes with the biomarkers of glucose metabolism

		The first season (winter)^a^				The four seasons^a^			
		*β*	95% CI		*p*^b^	*β*	95% CI		*p*^b^
			2.5%	97.5%			2.5%	97.5%	
Fasting blood glucose									
Green tea		6.20 × 10^−3^	− 2.89 × 10^−3^	1.53 × 10^−2^	0.18	− 4.89 × 10^−3^	− 9.64 × 10^−3^	− 1.33 × 10^−4^	**0.04**
Season	Spring					− 3.38 × 10^−1^	− 1.98	1.30	0.69
(Ref: winter)	Summer					− 3.81 × 10^−1^	− 2.05	1.29	0.65
	Autumn					− 1.78	− 3.46	− 1.01 × 10^−1^	**0.04**
Catechins		7.75 × 10^−3^	− 1.12 × 10^−2^	2.67 × 10^−2^	0.42	− 8.05 × 10^−4^	− 9.58 × 10^−3^	7.97 × 10^−3^	0.86
Season	Spring					− 1.54 × 10^−1^	− 1.80	1.49	0.85
(Ref: winter)	Summer					− 1.28 × 10^−1^	− 1.81	1.56	0.88
	Autumn					− 1.61	− 3.31	8.95 × 10^−2^	0.06
EGCG		2.32 × 10^−2^	− 3.36 × 10^−2^	8.01 × 10^−2^	0.42	4.23 × 10^−3^	− 2.52 × 10^−2^	3.36 × 10^−2^	0.78
Season	Spring					− 1.27 × 10^−1^	− 1.79	1.53	0.88
(Ref: winter)	Summer					− 1.21 × 10^−1^	− 1.81	1.57	0.89
	Autumn					− 1.65	− 3.36	5.06 × 10^−2^	0.06
HbA1c									
Green tea		7.57 × 10^−6^	− 2.74 × 10^−4^	2.89 × 10^−4^	0.96	9.82 × 10^−5^	− 5.00 × 10^−5^	2.46 × 10^−4^	0.19
Season	Spring					6.83 × 10^−3^	− 3.57 × 10^−2^	4.93 × 10^−2^	0.75
(Ref: winter)	Summer					− 2.06 × 10^−2^	− 6.53 × 10^−2^	2.42 × 10^−2^	0.37
	Autumn					− 1.98 × 10^−2^	− 6.59 × 10^−2^	2.63 × 10^−2^	0.40
Catechins		− 5.55 × 10^−4^	− 1.13 × 10^−3^	2.21 × 10^−5^	0.06	9.79 × 10^−5^	− 1.57 × 10^−4^	3.53 × 10^−4^	0.45
Season	Spring					3.33 × 10^−3^	− 3.90 × 10^−2^	4.57 × 10^−2^	0.88
(Ref: winter)	Summer					− 2.59 × 10^−2^	− 7.11 × 10^−2^	1.94 × 10^−2^	0.26
	Autumn					− 2.46 × 10^−2^	− 7.07 × 10^−2^	2.16 × 10^−2^	0.30
EGCG		− 1.65 × 10^−3^	− 3.39 × 10^−3^	8.06 × 10^−5^	0.06	1.76 × 10^−4^	− 6.28 × 10^−4^	9.80 × 10^−4^	0.67
Season	Spring					3.97 × 10^−3^	− 3.86 × 10^−2^	4.66 × 10^−2^	0.85
(Ref: winter)	Summer					− 2.53 × 10^−2^	− 7.06 × 10^−2^	1.99 × 10^−2^	0.27
	Autumn					− 2.41 × 10^−2^	− 7.03 × 10^−2^	2.21 × 10^−2^	0.30
Insulin									
Green tea		2.83 × 10^−4^	− 2.50 × 10^−4^	8.15 × 10^−4^	0.29	− 4.43 × 10^−4^	− 7.98 × 10^−4^	− 8.82 × 10^−5^	**0.01**
Season	Spring					− 2.56 × 10^−2^	− 1.59 × 10^−1^	1.08 × 10^−1^	0.71
(Ref: winter)	Summer					− 7.68 × 10^−2^	− 2.11 × 10^−1^	5.75 × 10^−2^	0.26
	Autumn					− 3.66 × 10^−2^	− 1.74 × 10^−1^	1.01 × 10^−1^	0.60
Catechins		6.74 × 10^−5^	− 1.03 × 10^−3^	1.16 × 10^−3^	0.90	− 4.53 × 10^−4^	− 1.17 × 10^−3^	2.64 × 10^−4^	0.21
Season	Spring					− 1.01 × 10^−2^	− 1.45 × 10^−1^	1.25 × 10^−1^	0.88
(Ref: winter)	Summer					− 5.27 × 10^−2^	− 1.88 × 10^−1^	8.24 × 10^−2^	0.44
	Autumn					− 1.43 × 10^−2^	− 1.55 × 10^−1^	1.27 × 10^−1^	0.84
EGCG		1.53 × 10^−4^	− 3.15 × 10^−3^	3.45 × 10^−3^	0.93	− 7.06 × 10^−4^	− 2.93 × 10^−3^	1.52 × 10^−3^	0.53
Season	Spring					− 1.14 × 10^−2^	− 1.48 × 10^−1^	1.26 × 10^−1^	0.87
(Ref: winter)	Summer					− 5.49 × 10^−2^	− 1.91 × 10^−1^	8.11 × 10^−2^	0.43
	Autumn					− 1.75 × 10^−2^	− 1.59 × 10^−1^	1.24 × 10^−1^	0.81
HOMA-IR									
Green tea		3.36 × 10^−4^	− 2.15 × 10^−4^	8.87 × 10^−4^	0.27	− 1.17 × 10^−4^	− 5.55 × 10^−4^	3.21 × 10^−4^	0.60
Season	Spring					− 1.69 × 10^−2^	− 1.58 × 10^−1^	1.24 × 10^−1^	0.81
(Ref: winter)	Summer					− 7.53 × 10^−2^	− 2.17 × 10^−1^	6.65 × 10^−2^	0.30
	Autumn					− 3.06 × 10^−2^	− 1.80 × 10^−1^	1.19 × 10^−1^	0.69
Catechins		1.49 × 10^−4^	− 9.85 × 10^−4^	1.28 × 10^−3^	0.79	− 5.90 × 10^−5^	− 8.16 × 10^−4^	6.98 × 10^−4^	0.88
Season	Spring					− 1.25 × 10^−2^	− 1.52 × 10^−1^	1.28 × 10^−1^	0.86
(Ref: winter)	Summer					− 6.89 × 10^−2^	− 2.08 × 10^−1^	7.04 × 10^−2^	0.33
	Autumn					− 2.52 × 10^−2^	− 1.72 × 10^−1^	1.22 × 10^−1^	0.74
EGCG		4.01 × 10^−4^	− 3.00 × 10^−3^	3.80 × 10^−3^	0.81	8.41 × 10^−4^	− 1.87 × 10^−3^	3.55 × 10^−3^	0.54
Season	Spring					− 7.52 × 10^−3^	− 1.48 × 10^−1^	1.33 × 10^−1^	0.92
(Ref: winter)	Summer					− 6.80 × 10^−2^	− 2.07 × 10^−1^	7.08 × 10^−2^	0.34
	Autumn					− 3.29 × 10^−2^	− 1.79 × 10^−1^	1.13 × 10^−1^	0.66

CI, confidence interval; EGCG, epigallocatechin-3-gallate; HbA1c, glycated hemoglobin A1c; HOMA-IR, homeostatic model assessment index of insulin resistance

^a^Beta coefficients, confidence intervals, and *p* values from the linear regression model for the first season and from the linear mixed-effects model for the four seasons

^b^*p* values shown in bold are statistically significant (*p* < 0.05)

### Associations of green tea and catechin intakes with the relative abundance of the gut microbiota

**Fig. 2:**
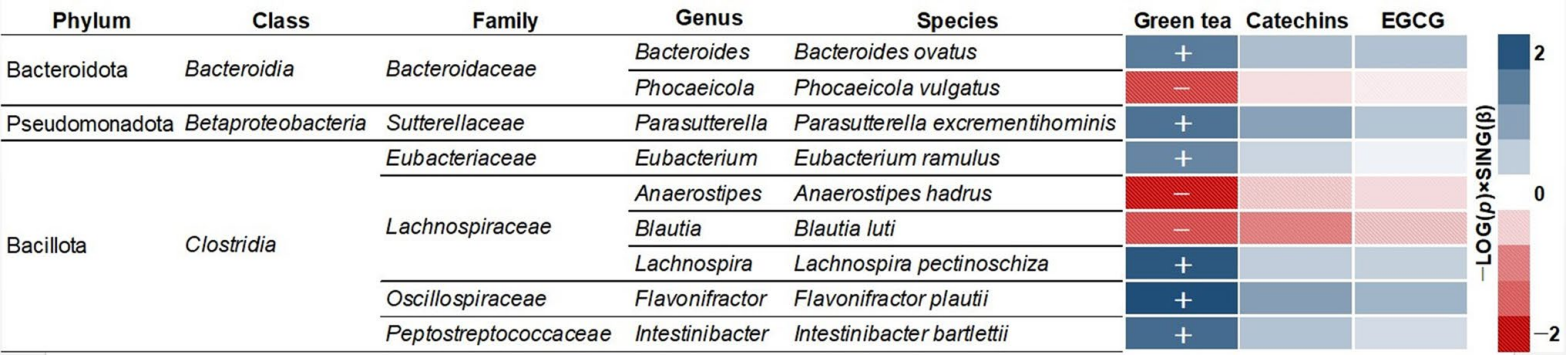
Heat map of the associations of green tea and catechin intakes with the relative abundances of the gut microbial species. Of the 71 species selected as shown in Table 3, only those significantly associated with green tea intake were plotted, with darker colors indicating smaller *p* values. Symbols represent significant positive (blue, +) and negative (red, −) associations, even after adjustment of *p* values for the false discovery rate (*q* < 0.20). Abbreviation: EGCG, epigallocatechin-3-gallate (color figure online)

At the phylum and genus levels, no association was observed between green tea intake and relative abundance of the gut microbiota (*p* > 0.05; Table 3). At the species level, green tea intake was positively associated with the relative abundances of *Bacteroides ovatus*, *Eubacterium ramulus*, *Intestinibacter bartlettii* (formerly *Clostridium bartlettii*), *Flavonifractor plautii*, *Lachnospira pectinoschiza*, and *Parasutterella excrementihominis*, but was negatively associated with the relative abundances of *Anaerostipes hadrus* (formerly *Eubacterium hadrum*), *P. vulgatus*, and *Blautia luti* (Fig. 2). No significant associations were observed between catechins intake and relative abundances of the gut microbiota (*p* > 0.20; Table 3) and between daily intakes of green tea and catechins and alpha-diversity (green tea: *β* = 4.67 × 10^−6^ [95% CI, − 2.11 × 10^−4^ to 2.20 × 10^–4^], *p* = 0.97; catechins: *β* = 2.81 × 10^−5^ [95% CI, − 3.12 × 10^−4^ to 3.68 × 10^−4^], *p* = 0.87; EGCG: *β* = 1.19 × 10^−4^ [95% CI, − 1.19 × 10^−3^ to 1.43 × 10^−3^], *p* = 0.86).

**Table 3:** Associations of green tea and catechin intakes with the relative abundance of the gut microbiota

		Green tea intake^a^					Catechins intake^a^					EGCG intake^a^				
		*β*	95% CI		*p*	*q*^b^	*β*	95% CI		*p*	*q*^b^	*β*	95% CI		*p*	*q*^b^
			2.5%	97.5%				2.5%	97.5%				2.5%	97.5%		
Phylum	Actinomycetota	− 4.95 × 10^−5^	− 6.53 × 10^−4^	5.54 × 10^−4^	0.87	0.99	1.38 × 10^−4^	− 9.54 × 10^−4^	1.23 × 10^−3^	0.80	0.99	8.14 × 10^−4^	− 2.81 × 10^−3^	4.44 × 10^−3^	0.66	0.99
	Bacillota	− 2.23 × 10^−5^	− 4.96 × 10^−4^	4.51 × 10^−4^	0.93	0.99	− 3.18 × 10^−5^	− 9.55 × 10^−4^	8.91 × 10^−4^	0.95	0.99	1.80 × 10^−4^	− 2.85 × 10^−3^	3.21 × 10^−3^	0.91	0.99
	Bacteroidota	− 8.48 × 10^−5^	− 6.24 × 10^−4^	4.55 × 10^−4^	0.76	0.99	3.74 × 10^−6^	− 1.01 × 10^−3^	1.02 × 10^−3^	0.99	0.99	− 1.67 × 10^−5^	− 3.43 × 10^−3^	3.40 × 10^−3^	0.99	0.99
	Fusobacteria	1.14 × 10^−3^	− 1.78 × 10^−4^	2.46 × 10^−3^	0.09	0.54	2.42 × 10^−3^	− 2.84 × 10^−4^	5.12 × 10^−3^	0.08	0.48	6.98 × 10^−3^	− 2.41 × 10^−3^	1.64 × 10^−2^	0.14	0.54
	Pseudomonadota	5.87 × 10^−6^	− 6.28 × 10^−4^	6.40 × 10^−4^	0.99	0.99	− 5.15 × 10^−4^	− 1.71 × 10^−3^	6.77 × 10^−4^	0.40	0.79	− 2.62 × 10^−3^	− 6.46 × 10^−3^	1.22 × 10^−3^	0.18	0.54
	Verrucomicrobia	− 3.55 × 10^−4^	− 1.83 × 10^−3^	1.12 × 10^−3^	0.64	0.99	− 1.29 × 10^−3^	− 4.26 × 10^−3^	1.68 × 10^−3^	0.39	0.79	− 4.52 × 10^−3^	− 1.47 × 10^−2^	5.64 × 10^−3^	0.38	0.76
Genus	*Agathobacter*	− 7.98 × 10^−5^	− 1.14 × 10^−3^	9.79 × 10^−4^	0.88	0.91	− 6.26 × 10^−4^	− 2.57 × 10^−3^	1.32 × 10^−3^	0.53	0.51	− 1.76 × 10^−3^	− 8.32 × 10^−3^	4.81 × 10^−3^	0.60	0.98
	*Akkermansia*	6.28 × 10^−5^	− 1.19 × 10^−3^	1.32 × 10^−3^	0.92	0.93	− 8.42 × 10^−4^	− 3.32 × 10^−3^	1.63 × 10^−3^	0.50	0.51	− 3.68 × 10^−3^	− 1.22 × 10^−2^	4.83 × 10^−3^	0.40	0.98
	*Alistipes*	− 4.77 × 10^−4^	− 1.48 × 10^−3^	5.26 × 10^−4^	0.35	0.64	− 4.74 × 10^−4^	− 2.21 × 10^−3^	1.27 × 10^−3^	0.59	0.51	− 1.58 × 10^−3^	− 7.31 × 10^−3^	4.15 × 10^−3^	0.59	0.98
	*Anaerobutyricum*	− 4.87 × 10^−4^	− 1.34 × 10^−3^	3.67 × 10^−4^	0.26	0.59	− 2.46 × 10^−4^	− 1.71 × 10^−3^	1.22 × 10^−3^	0.74	0.54	− 4.68 × 10^−4^	− 5.54 × 10^−3^	4.60 × 10^−3^	0.86	0.98
	*Anaerostipes*	− 7.78 × 10^−4^	− 1.67 × 10^−3^	1.17 × 10^−4^	0.09	0.48	− 5.68 × 10^−4^	− 2.17 × 10^−3^	1.03 × 10^−3^	0.48	0.51	− 9.89 × 10^−4^	− 6.42 × 10^−3^	4.44 × 10^−3^	0.72	0.98
	*Bacteroides*	1.41 × 10^−4^	− 5.14 × 10^−4^	7.96 × 10^−4^	0.67	0.80	4.36 × 10^−4^	− 7.31 × 10^−4^	1.60 × 10^−3^	0.46	0.51	1.65 × 10^−3^	− 2.19 × 10^−3^	5.48 × 10^−3^	0.40	0.98
	*Barnesiella*	− 6.11 × 10^−4^	− 1.47 × 10^−3^	2.50 × 10^−4^	0.16	0.50	4.45 × 10^−4^	− 1.25 × 10^−3^	2.14 × 10^−3^	0.60	0.51	1.23 × 10^−3^	− 4.58 × 10^−3^	7.03 × 10^−3^	0.68	0.98
	*Bifidobacterium*	− 3.38 × 10^−5^	− 1.04 × 10^−3^	9.76 × 10^−4^	0.95	0.93	− 4.53 × 10^−4^	− 2.23 × 10^−3^	1.33 × 10^−3^	0.62	0.51	− 1.06 × 10^−3^	− 7.13 × 10^−3^	5.01 × 10^−3^	0.73	0.98
	*Bilophila*	2.81 × 10^−4^	− 4.76 × 10^−4^	1.04 × 10^−3^	0.47	0.64	8.13 × 10^−5^	− 1.40 × 10^−3^	1.56 × 10^−3^	0.91	0.57	− 5.22 × 10^−4^	− 5.68 × 10^−3^	4.63 × 10^−3^	0.84	0.98
	*Blautia*	− 2.45 × 10^−4^	− 8.68 × 10^−4^	3.79 × 10^−4^	0.44	0.64	9.72 × 10^−5^	− 9.69 × 10^−4^	1.16 × 10^−3^	0.86	0.56	7.97 × 10^−4^	− 2.86 × 10^−3^	4.45 × 10^−3^	0.67	0.98
	*Clostridium*	1.30 × 10^−4^	− 8.29 × 10^−4^	1.09 × 10^−3^	0.79	0.86	− 1.96 × 10^−4^	− 1.91 × 10^−3^	1.52 × 10^−3^	0.82	0.56	− 7.29 × 10^−4^	− 6.49 × 10^−3^	5.03 × 10^−3^	0.80	0.98
	*Collinsella*	− 2.99 × 10^−4^	− 1.25 × 10^−3^	6.56 × 10^−4^	0.54	0.69	3.64 × 10^−4^	− 1.30 × 10^−3^	2.03 × 10^−3^	0.67	0.53	2.52 × 10^−3^	− 2.70 × 10^−3^	7.74 × 10^−3^	0.34	0.98
	*Coprococcus*	6.05 × 10^−6^	− 1.08 × 10^−3^	1.09 × 10^−3^	0.99	0.93	− 7.71 × 10^−4^	− 2.68 × 10^−3^	1.14 × 10^−3^	0.43	0.51	− 1.22 × 10^−4^	− 6.22 × 10^−3^	5.97 × 10^−3^	0.97	0.99
	*Dialister*	− 1.27 × 10^−3^	− 2.58 × 10^−3^	4.29 × 10^−5^	0.06	0.48	− 1.83 × 10^−3^	− 4.31 × 10^−3^	6.47 × 10^−4^	0.15	0.51	− 4.68 × 10^−3^	− 1.27 × 10^−2^	3.35 × 10^−3^	0.25	0.98
	*Dorea*	− 7.06 × 10^−4^	− 1.65 × 10^−3^	2.42 × 10^−4^	0.14	0.50	− 9.14 × 10^−4^	− 2.57 × 10^−3^	7.42 × 10^−4^	0.28	0.51	− 1.27 × 10^−3^	− 6.49 × 10^−3^	3.96 × 10^−3^	0.63	0.98
	*Eggerthella*	1.28 × 10^−4^	− 7.26 × 10^−4^	9.83 × 10^−4^	0.77	0.86	1.21 × 10^−3^	− 3.95 × 10^−4^	2.82 × 10^−3^	0.14	0.51	4.27 × 10^−3^	− 1.43 × 10^−3^	9.96 × 10^−3^	0.14	0.96
	*Enterocloster*	3.16 × 10^−4^	− 4.72 × 10^−4^	1.10 × 10^−3^	0.43	0.64	6.09 × 10^−4^	− 8.25 × 10^−4^	2.04 × 10^−3^	0.40	0.51	1.02 × 10^−3^	− 3.67 × 10^−3^	5.71 × 10^−3^	0.67	0.98
	*Eubacterium*	− 5.34 × 10^−4^	− 1.43 × 10^−3^	3.67 × 10^−4^	0.24	0.59	− 8.24 × 10^−5^	− 1.66 × 10^−3^	1.50 × 10^−3^	0.92	0.57	− 4.54 × 10^−4^	− 5.66 × 10^−3^	4.75 × 10^−3^	0.86	0.98
	*Faecalibacterium*	6.98 × 10^−5^	− 6.61 × 10^−4^	8.00 × 10^−4^	0.85	0.90	2.66 × 10^−4^	− 1.09 × 10^−3^	1.62 × 10^−3^	0.70	0.54	7.57 × 10^−4^	− 3.74 × 10^−3^	5.25 × 10^−3^	0.74	0.98
	*Fusobacterium*	1.16 × 10^−3^	− 9.41 × 10^−5^	2.41 × 10^−3^	0.07	0.48	1.51 × 10^−3^	− 9.82 × 10^−4^	4.00 × 10^−3^	0.23	0.51	4.26 × 10^−3^	− 4.02 × 10^−3^	1.26 × 10^−2^	0.31	0.98
	*Intestinibacter*	1.39 × 10^−3^	2.89 × 10^−4^	2.49 × 10^−3^	0.01	0.44	1.46 × 10^−3^	− 6.35 × 10^−4^	3.56 × 10^−3^	0.17	0.51	2.92 × 10^−3^	− 4.12 × 10^−3^	9.97 × 10^−3^	0.41	0.98
	*Lachnospira*	1.10 × 10^−3^	4.40 × 10^−5^	2.16 × 10^−3^	0.04	0.48	6.29 × 10^−4^	− 1.40 × 10^−3^	2.66 × 10^−3^	0.54	0.51	8.96 × 10^−4^	− 5.93 × 10^−3^	7.72 × 10^−3^	0.80	0.98
	*Lacrimispora*	− 6.09 × 10^−4^	− 1.51 × 10^−3^	2.90 × 10^−4^	0.18	0.50	− 9.24 × 10^−4^	− 2.57 × 10^−3^	7.18 × 10^−4^	0.27	0.51	− 2.00 × 10^−3^	− 7.37 × 10^−3^	3.36 × 10^−3^	0.46	0.98
	*Mediterraneibacter*	2.63 × 10^−4^	− 7.97 × 10^−4^	1.32 × 10^−3^	0.62	0.77	9.71 × 10^−4^	− 8.71 × 10^−4^	2.81 × 10^−3^	0.30	0.51	2.59 × 10^−3^	− 3.74 × 10^−3^	8.93 × 10^−3^	0.42	0.98
	*Megamonas*	− 7.27 × 10^−4^	− 2.42 × 10^−3^	9.60 × 10^−4^	0.40	0.64	− 3.04 × 10^−4^	− 3.45 × 10^−3^	2.84 × 10^−3^	0.85	0.56	− 2.66 × 10^−3^	− 1.30 × 10^−2^	7.68 × 10^−3^	0.61	0.98
	*Odoribacter*	3.00 × 10^−4^	− 4.04 × 10^−4^	1.00 × 10^−3^	0.40	0.64	4.47 × 10^−4^	− 9.25 × 10^−4^	1.82 × 10^−3^	0.52	0.51	6.55 × 10^−4^	− 4.12 × 10^−3^	5.43 × 10^−3^	0.79	0.98
	*Parabacteroides*	− 4.63 × 10^−4^	− 1.32 × 10^−3^	3.90 × 10^−4^	0.29	0.61	4.60 × 10^−4^	− 1.10 × 10^−3^	2.02 × 10^−3^	0.56	0.51	2.85 × 10^−3^	− 2.44 × 10^−3^	8.14 × 10^−3^	0.29	0.98
	*Parasutterella*	7.74 × 10^−4^	− 2.47 × 10^−4^	1.79 × 10^−3^	0.14	0.50	7.15 × 10^−4^	− 1.19 × 10^−3^	2.62 × 10^−3^	0.46	0.51	1.48 × 10^−4^	− 6.22 × 10^−3^	6.52 × 10^−3^	0.96	0.99
	*Phascolarctobacterium*	5.26 × 10^−4^	− 5.00 × 10^−4^	1.55 × 10^−3^	0.31	0.63	1.53 × 10^−3^	− 3.11 × 10^−4^	3.38 × 10^−3^	0.10	0.51	5.24 × 10^−3^	− 1.02 × 10^−3^	1.15 × 10^−2^	0.10	0.96
	*Phocaeicola*	5.43 × 10^−4^	− 2.07 × 10^−4^	1.29 × 10^−3^	0.15	0.50	1.20 × 10^−3^	− 5.85 × 10^−5^	2.45 × 10^−3^	0.06	0.51	3.52 × 10^−3^	− 7.45 × 10^−4^	7.78 × 10^−3^	0.11	0.96
	*Prevotella*	5.07 × 10^−6^	− 1.14 × 10^−3^	1.15 × 10^−3^	0.99	0.93	− 1.21 × 10^−3^	− 3.56 × 10^−3^	1.13 × 10^−3^	0.31	0.51	− 5.24 × 10^−4^	− 8.88 × 10^−3^	7.83 × 10^−3^	0.90	0.98
	*Romboutsia*	3.93 × 10^−4^	− 6.66 × 10^−4^	1.45 × 10^−3^	0.47	0.64	1.14 × 10^−3^	− 8.80 × 10^−4^	3.15 × 10^−3^	0.27	0.51	3.99 × 10^−3^	− 2.93 × 10^−3^	1.09 × 10^−2^	0.26	0.98
	*Roseburia*	3.70 × 10^−4^	− 5.33 × 10^−4^	1.27 × 10^−3^	0.42	0.64	2.72 × 10^−4^	− 1.40 × 10^−3^	1.94 × 10^−3^	0.75	0.54	4.07 × 10^−5^	− 5.43 × 10^−3^	5.51 × 10^−3^	0.99	0.99
	*Ruminococcus*	7.49 × 10^−4^	1.04 × 10^−4^	1.39 × 10^−3^	0.02	0.44	8.87 × 10^−4^	− 2.61 × 10^−4^	2.04 × 10^−3^	0.13	0.51	2.96 × 10^−3^	− 9.57 × 10^−4^	6.88 × 10^−3^	0.14	0.96
	*Segatella*	5.68 × 10^−4^	− 1.03 × 10^−3^	2.17 × 10^−3^	0.48	0.64	1.91 × 10^−3^	− 9.12 × 10^−4^	4.73 × 10^−3^	0.18	0.51	3.06 × 10^−3^	− 6.11 × 10^−3^	1.22 × 10^−2^	0.51	0.98
	*Sphingomonas*	− 4.64 × 10^−4^	− 1.42 × 10^−3^	4.93 × 10^−4^	0.34	0.64	− 2.02 × 10^−3^	− 4.03 × 10^−3^	− 1.14 × 10^−5^	0.05	0.51	− 7.49 × 10^−3^	− 1.46 × 10^−2^	− 4.16 × 10^−4^	0.04	0.78
	*Streptococcus*	1.73 × 10^−4^	− 8.27 × 10^−4^	1.17 × 10^−3^	0.73	0.85	3.42 × 10^−5^	− 1.75 × 10^−3^	1.82 × 10^−3^	0.97	0.58	− 5.74 × 10^−4^	− 6.47 × 10^−3^	5.32 × 10^−3^	0.85	0.98
	*Subdoligranulum*	8.12 × 10^−4^	− 1.21 × 10^−4^	1.75 × 10^−3^	0.09	0.48	4.37 × 10^−4^	− 1.26 × 10^−3^	2.13 × 10^−3^	0.61	0.51	1.44 × 10^−3^	− 4.11 × 10^−3^	6.98 × 10^−3^	0.61	0.98
	*Sutterella*	− 8.62 × 10^−4^	− 1.93 × 10^−3^	2.05 × 10^−4^	0.11	0.50	− 1.67 × 10^−3^	− 3.57 × 10^−3^	2.39 × 10^−4^	0.09	0.51	− 7.10 × 10^−3^	− 1.38 × 10^−2^	− 4.50 × 10^−4^	0.04	0.78
	*Thomasclavelia*	6.24 × 10^−4^	− 4.39 × 10^−4^	1.69 × 10^−3^	0.25	0.59	− 9.20 × 10^−4^	− 2.98 × 10^−3^	1.14 × 10^−3^	0.38	0.51	− 3.32 × 10^−3^	− 1.03 × 10^−2^	3.67 × 10^−3^	0.35	0.98
	*Veillonella*	9.77 × 10^−4^	− 4.25 × 10^−4^	2.38 × 10^−3^	0.17	0.50	2.79 × 10^−4^	− 2.21 × 10^−3^	2.77 × 10^−3^	0.83	0.56	4.83 × 10^−4^	− 7.60 × 10^−3^	8.56 × 10^−3^	0.91	0.98
	*Blautia wexlerae*	− 4.53 × 10^−4^	− 1.26 × 10^−3^	3.51 × 10^−4^	0.27	0.41	− 2.62 × 10^−4^	− 1.72 × 10^−3^	1.20 × 10^−3^	0.72	0.99	− 1.06 × 10^−4^	− 5.00 × 10^−3^	4.79 × 10^−3^	0.97	0.99
	*Faecalibacterium prausnitzii*	− 5.32 × 10^−5^	− 7.69 × 10^−4^	6.63 × 10^−4^	0.88	0.49	1.56 × 10^−4^	− 1.20 × 10^−3^	1.52 × 10^−3^	0.82	0.99	7.87 × 10^−4^	− 3.66 × 10^−3^	5.24 × 10^−3^	0.73	0.98
	*Streptococcus salivarius*	2.32 × 10^−4^	− 7.95 × 10^−4^	1.26 × 10^−3^	0.66	0.48	6.67 × 10^−4^	− 1.13 × 10^−3^	2.47 × 10^−3^	0.47	0.98	1.26 × 10^−3^	− 4.75 × 10^−3^	7.26 × 10^−3^	0.68	0.98
	*Bacteroides uniformis*	3.58 × 10^−4^	− 5.04 × 10^−4^	1.22 × 10^−3^	0.41	0.45	4.50 × 10^−4^	− 1.06 × 10^−3^	1.96 × 10^−3^	0.56	0.98	1.77 × 10^−3^	− 3.23 × 10^−3^	6.78 × 10^−3^	0.49	0.98
	*Blautia faecis*	5.57 × 10^−5^	− 5.78 × 10^−4^	6.89 × 10^−4^	0.86	0.49	− 5.71 × 10^−4^	− 1.73 × 10^−3^	5.90 × 10^−4^	0.33	0.95	− 2.28 × 10^−3^	− 6.25 × 10^−3^	1.69 × 10^−3^	0.26	0.98
	*Blautia luti*	− 1.14 × 10^−3^	− 2.21 × 10^−3^	− 7.68 × 10^−5^	**0.04**	**0.17**	− 1.59 × 10^−3^	− 3.47 × 10^−3^	2.87 × 10^−4^	0.10	0.76	− 3.32 × 10^−3^	− 9.53 × 10^−3^	2.88 × 10^−3^	0.29	0.98
	*Anaerostipes hadrus*	− 1.27 × 10^−3^	− 2.26 × 10^−3^	− 2.86 × 10^−4^	**0.01**	**0.16**	− 8.87 × 10^−4^	− 2.72 × 10^−3^	9.49 × 10^−4^	0.34	0.95	− 2.00 × 10^−3^	− 8.18 × 10^−3^	4.19 × 10^−3^	0.53	0.98
	*Bifidobacterium longum*	− 3.76 × 10^−5^	− 1.06 × 10^−3^	9.90 × 10^−4^	0.94	0.50	− 2.70 × 10^−4^	− 2.11 × 10^−3^	1.57 × 10^−3^	0.77	0.99	− 1.94 × 10^−4^	− 6.09 × 10^−3^	5.70 × 10^−3^	0.95	0.99
	*Collinsella aerofaciens*	− 8.30 × 10^−4^	− 1.77 × 10^−3^	1.07 × 10^−4^	0.08	0.21	1.43 × 10^−4^	− 1.48 × 10^−3^	1.77 × 10^−3^	0.86	0.99	1.32 × 10^−3^	− 3.96 × 10^−3^	6.61 × 10^−3^	0.62	0.98
	*Subdoligranulum variabile*	6.52 × 10^−4^	− 3.32 × 10^−4^	1.64 × 10^−3^	0.19	0.33	2.28 × 10^−4^	− 1.45 × 10^−3^	1.90 × 10^−3^	0.79	0.99	1.28 × 10^−3^	− 4.18 × 10^−3^	6.73 × 10^−3^	0.64	0.98
	*Anaerobutyricum hallii*	− 7.93 × 10^−4^	− 1.71 × 10^−3^	1.27 × 10^−4^	0.09	0.21	− 5.30 × 10^−4^	− 2.06 × 10^−3^	1.00 × 10^−3^	0.50	0.98	− 1.20 × 10^−3^	− 6.63 × 10^−3^	4.23 × 10^−3^	0.66	0.98
	*Ruminococcus gnavus*	1.03 × 10^−3^	− 1.83 × 10^−4^	2.24 × 10^−3^	0.10	0.21	2.73 × 10^−3^	5.06 × 10^−4^	4.96 × 10^−3^	0.02	0.72	6.65 × 10^−3^	− 8.58 × 10^−4^	1.42 × 10^−2^	0.08	0.98
	*Phocaeicola vulgatus*	− 1.39 × 10^−3^	− 2.63 × 10^−3^	− 1.54 × 10^−4^	**0.03**	**0.17**	− 6.32 × 10^−4^	− 2.87 × 10^−3^	1.60 × 10^−3^	0.58	0.98	− 8.81 × 10^−4^	− 8.09 × 10^−3^	6.33 × 10^−3^	0.81	0.98
	*Phocaeicola dorei*	1.14 × 10^−3^	− 1.77 × 10^−4^	2.45 × 10^−3^	0.09	0.21	1.97 × 10^−3^	− 2.58 × 10^−4^	4.20 × 10^−3^	0.08	0.76	5.60 × 10^−3^	− 1.79 × 10^−3^	1.30 × 10^−2^	0.14	0.98
	*Blautia obeum*	− 3.22 × 10^−4^	− 1.15 × 10^−3^	5.09 × 10^−4^	0.45	0.45	4.02 × 10^−5^	− 1.44 × 10^−3^	1.52 × 10^−3^	0.96	0.99	4.06 × 10^−4^	− 4.68 × 10^−3^	5.49 × 10^−3^	0.88	0.98
	*Mediterraneibacter faecis*	1.83 × 10^−4^	− 1.02 × 10^−3^	1.39 × 10^−3^	0.76	0.48	1.25 × 10^−3^	− 8.18 × 10^−4^	3.31 × 10^−3^	0.24	0.82	2.42 × 10^−3^	− 4.32 × 10^−3^	9.17 × 10^−3^	0.48	0.98
	*Bacteroides xylanisolvens*	− 1.51 × 10^−4^	− 1.05 × 10^−3^	7.47 × 10^−4^	0.74	0.48	2.06 × 10^−4^	− 1.33 × 10^−3^	1.75 × 10^−3^	0.79	0.99	1.67 × 10^−3^	− 3.50 × 10^−3^	6.85 × 10^−3^	0.52	0.98
	*Sphingomonas leidyi*	− 5.67 × 10^−4^	− 1.51 × 10^−3^	3.77 × 10^−4^	0.24	0.39	− 2.13 × 10^−3^	− 4.05 × 10^−3^	− 2.03 × 10^−4^	0.03	0.72	− 7.52 × 10^−3^	− 1.43 × 10^−2^	− 7.09 × 10^−4^	0.03	0.98
	*Bacteroides ovatus*	8.95 × 10^−4^	7.13 × 10^−5^	1.72 × 10^−3^	**0.03**	**0.17**	1.01 × 10^−3^	− 5.03 × 10^−4^	2.52 × 10^−3^	0.19	0.82	3.21 × 10^−3^	− 1.83 × 10^−3^	8.26 × 10^−3^	0.21	0.98
	*Flavonifractor plautii*	9.62 × 10^−4^	2.34 × 10^−4^	1.69 × 10^−3^	**0.01**	**0.16**	1.19 × 10^−3^	− 1.67 × 10^−4^	2.55 × 10^−3^	0.09	0.76	3.26 × 10^−3^	− 1.18 × 10^−3^	7.71 × 10^−3^	0.15	0.98
	*Ruminococcus torques*	− 3.95 × 10^−4^	− 1.45 × 10^−3^	6.60 × 10^−4^	0.46	0.45	− 4.69 × 10^−4^	− 2.32 × 10^−3^	1.38 × 10^−3^	0.62	0.99	− 6.07 × 10^−4^	− 6.83 × 10^−3^	5.61 × 10^−3^	0.85	0.98
	*Bacteroides faecichinchillae*	− 1.73 × 10^−4^	− 1.08 × 10^−3^	7.30 × 10^−4^	0.71	0.48	5.22 × 10^−4^	− 1.07 × 10^−3^	2.12 × 10^−3^	0.52	0.98	1.14 × 10^−3^	− 3.99 × 10^−3^	6.27 × 10^−3^	0.66	0.98
	*Dorea longicatena*	− 7.31 × 10^−4^	− 1.70 × 10^−3^	2.32 × 10^−4^	0.14	0.25	− 1.46 × 10^−3^	− 3.15 × 10^−3^	2.40 × 10^−4^	0.09	0.76	− 2.81 × 10^−3^	− 8.41 × 10^−3^	2.79 × 10^−3^	0.32	0.98
	*Roseburia faecis*	4.56 × 10^−4^	− 5.59 × 10^−4^	1.47 × 10^−3^	0.38	0.45	8.43 × 10^−5^	− 1.83 × 10^−3^	2.00 × 10^−3^	0.93	0.99	1.06 × 10^−4^	− 6.23 × 10^−3^	6.44 × 10^−3^	0.97	0.99
	*Lacrimispora xylanolytica*	1.57 × 10^−4^	− 7.84 × 10^−4^	1.10 × 10^−3^	0.74	0.48	− 1.15 × 10^−3^	− 2.98 × 10^−3^	6.83 × 10^−4^	0.22	0.82	− 2.45 × 10^−3^	− 8.46 × 10^−3^	3.56 × 10^−3^	0.42	0.98
	*Romboutsia lituseburensis*	2.00 × 10^−4^	− 8.75 × 10^−4^	1.28 × 10^−3^	0.71	0.48	7.76 × 10^−4^	− 1.26 × 10^−3^	2.81 × 10^−3^	0.45	0.98	3.58 × 10^−3^	− 3.29 × 10^−3^	1.05 × 10^−2^	0.31	0.98
	*Ruminococcus lactaris*	− 3.18 × 10^−4^	− 1.30 × 10^−3^	6.68 × 10^−4^	0.53	0.45	− 1.05 × 10^−3^	− 2.82 × 10^−3^	7.14 × 10^−4^	0.24	0.82	− 2.99 × 10^−3^	− 8.73 × 10^−3^	2.75 × 10^−3^	0.31	0.98
	*Parabacteroides distasonis*	− 2.58 × 10^−4^	− 1.14 × 10^−3^	6.26 × 10^−4^	0.57	0.45	2.86 × 10^−4^	− 1.36 × 10^−3^	1.93 × 10^−3^	0.73	0.99	8.99 × 10^−4^	− 4.54 × 10^−3^	6.34 × 10^−3^	0.75	0.98
	*Enterocloster bolteae*	8.21 × 10^−4^	− 5.01 × 10^−5^	1.69 × 10^−3^	0.06	0.20	1.85 × 10^−3^	1.90 × 10^−4^	3.52 × 10^−3^	0.03	0.72	4.75 × 10^−3^	− 7.48 × 10^−4^	1.02 × 10^−2^	0.09	0.98
	*Agathobacter rectalis*	− 1.13 × 10^−4^	− 1.17 × 10^−3^	9.46 × 10^−4^	0.83	0.49	− 6.39 × 10^−4^	− 2.58 × 10^−3^	1.30 × 10^−3^	0.52	0.98	− 1.41 × 10^−3^	− 7.76 × 10^−3^	4.95 × 10^−3^	0.66	0.98
	*Intestinibacter bartlettii*	1.28 × 10^−3^	1.89 × 10^−4^	2.38 × 10^−3^	**0.02**	**0.17**	1.25 × 10^−3^	− 7.57 × 10^−4^	3.27 × 10^−3^	0.22	0.82	2.76 × 10^−3^	− 3.91 × 10^−3^	9.42 × 10^−3^	0.42	0.98
	*Eggerthella lenta*	3.71 × 10^−4^	− 4.18 × 10^−4^	1.16 × 10^−3^	0.36	0.45	1.20 × 10^−3^	− 2.61 × 10^−4^	2.65 × 10^−3^	0.11	0.76	4.30 × 10^−3^	− 5.51 × 10^−4^	9.15 × 10^−3^	0.08	0.98
	*Streptococcus sinensis*	1.35 × 10^−4^	− 7.21 × 10^−4^	9.92 × 10^−4^	0.76	0.48	− 2.53 × 10^−5^	− 1.78 × 10^−3^	1.73 × 10^−3^	0.98	0.99	9.88 × 10^−4^	− 4.96 × 10^−3^	6.94 × 10^−3^	0.74	0.98
	*Segatella copri*	6.15 × 10^−4^	− 9.27 × 10^−4^	2.16 × 10^−3^	0.43	0.45	2.08 × 10^−3^	− 6.06 × 10^−4^	4.77 × 10^−3^	0.13	0.82	4.33 × 10^−3^	− 4.35 × 10^−3^	1.30 × 10^−2^	0.33	0.98
	*Phocaeicola plebeius*	3.77 × 10^−4^	− 1.12 × 10^−3^	1.87 × 10^−3^	0.62	0.47	1.72 × 10^−3^	− 9.60 × 10^−4^	4.39 × 10^−3^	0.21	0.82	3.36 × 10^−3^	− 5.28 × 10^−3^	1.20 × 10^−2^	0.44	0.98
	*Alistipes onderdonkii*	4.05 × 10^−4^	− 5.75 × 10^−4^	1.38 × 10^−3^	0.42	0.45	− 4.17 × 10^−5^	− 1.77 × 10^−3^	1.68 × 10^−3^	0.96	0.99	6.43 × 10^−4^	− 4.99 × 10^−3^	6.28 × 10^−3^	0.82	0.98
	*Eubacterium ventriosum*	2.27 × 10^−4^	− 5.20 × 10^−4^	9.75 × 10^−4^	0.55	0.45	8.51 × 10^−4^	− 6.21 × 10^−4^	2.32 × 10^−3^	0.26	0.83	2.27 × 10^−3^	− 2.70 × 10^−3^	7.24 × 10^−3^	0.37	0.98
	*Roseburia inulinivorans*	7.01 × 10^−4^	− 2.71 × 10^−4^	1.67 × 10^−3^	0.16	0.28	5.51 × 10^−4^	− 1.27 × 10^−3^	2.37 × 10^−3^	0.55	0.98	2.27 × 10^−5^	− 6.07 × 10^−3^	6.12 × 10^−3^	0.99	0.99
	*Ruminococcus bromii*	6.08 × 10^−4^	− 8.64 × 10^−4^	2.08 × 10^−3^	0.42	0.45	− 2.18 × 10^−4^	− 2.82 × 10^−3^	2.39 × 10^−3^	0.87	0.99	− 1.04 × 10^−3^	− 9.65 × 10^−3^	7.57 × 10^−3^	0.81	0.98
	*Bilophila wadsworthia*	6.03 × 10^−5^	− 5.96 × 10^−4^	7.17 × 10^−4^	0.86	0.49	− 7.87 × 10^−6^	− 1.27 × 10^−3^	1.25 × 10^−3^	0.99	0.99	− 1.89 × 10^−5^	− 4.18 × 10^−3^	4.14 × 10^−3^	0.99	0.99
	*Dorea formicigenerans*	− 1.81 × 10^−4^	− 1.02 × 10^−3^	6.59 × 10^−4^	0.67	0.48	3.81 × 10^−4^	− 1.15 × 10^−3^	1.91 × 10^−3^	0.62	0.99	9.21 × 10^−4^	− 4.24 × 10^−3^	6.08 × 10^−3^	0.73	0.98
	*Odoribacter splanchnicus*	1.88 × 10^−4^	− 4.30 × 10^−4^	8.06 × 10^−4^	0.55	0.45	4.28 × 10^−5^	− 1.19 × 10^−3^	1.28 × 10^−3^	0.95	0.99	− 6.35 × 10^−4^	− 4.96 × 10^−3^	3.70 × 10^−3^	0.77	0.98
	*Alistipes putredinis*	− 8.75 × 10^−4^	− 1.96 × 10^−3^	2.06 × 10^−4^	0.11	0.22	− 1.51 × 10^−3^	− 3.54 × 10^−3^	5.08 × 10^−4^	0.14	0.82	− 4.13 × 10^−3^	− 1.09 × 10^−2^	2.64 × 10^−3^	0.23	0.98
	*Megamonas funiformis*	− 4.79 × 10^−4^	− 2.12 × 10^−3^	1.16 × 10^−3^	0.57	0.45	− 1.21 × 10^−4^	− 3.11 × 10^−3^	2.87 × 10^−3^	0.94	0.99	− 2.08 × 10^−3^	− 1.18 × 10^−2^	7.67 × 10^−3^	0.67	0.98
	*Parabacteroides merdae*	− 9.84 × 10^−4^	− 2.13 × 10^−3^	1.67 × 10^−4^	0.09	0.21	− 4.61 × 10^−4^	− 2.55 × 10^−3^	1.62 × 10^−3^	0.66	0.99	− 7.74 × 10^−4^	− 7.74 × 10^−3^	6.19 × 10^−3^	0.83	0.98
	*Clostridium leptum*	4.53 × 10^−4^	− 4.99 × 10^−4^	1.41 × 10^−3^	0.35	0.45	1.17 × 10^−3^	− 6.82 × 10^−4^	3.02 × 10^−3^	0.21	0.82	2.86 × 10^−3^	− 3.61 × 10^−3^	9.33 × 10^−3^	0.38	0.98
	*Bacteroides stercoris*	− 1.79 × 10^−4^	− 1.44 × 10^−3^	1.08 × 10^−3^	0.78	0.48	− 9.97 × 10^−4^	− 3.25 × 10^−3^	1.26 × 10^−3^	0.38	0.97	− 3.70 × 10^−3^	− 1.10 × 10^−2^	3.56 × 10^−3^	0.32	0.98
	*Phocaeicola coprocola*	− 1.45 × 10^−4^	− 1.76 × 10^−3^	1.47 × 10^−3^	0.86	0.49	4.49 × 10^−4^	− 2.30 × 10^−3^	3.20 × 10^−3^	0.75	0.99	7.93 × 10^−4^	− 8.31 × 10^−3^	9.89 × 10^−3^	0.86	0.98
	*Coprococcus comes*	− 1.06 × 10^−4^	− 1.15 × 10^−3^	9.32 × 10^−4^	0.84	0.49	− 7.14 × 10^−4^	− 2.53 × 10^−3^	1.10 × 10^−3^	0.44	0.98	− 1.11 × 10^−4^	− 5.97 × 10^−3^	5.75 × 10^−3^	0.97	0.99
	*Bacteroides faecis*	− 3.15 × 10^−4^	− 1.29 × 10^−3^	6.64 × 10^−4^	0.53	0.45	− 8.25 × 10^−4^	− 2.61 × 10^−3^	9.62 × 10^−4^	0.36	0.96	− 2.16 × 10^−3^	− 8.04 × 10^−3^	3.72 × 10^−3^	0.47	0.98
	*Blautia glucerasea*	5.90 × 10^−5^	− 1.13 × 10^−3^	1.25 × 10^−3^	0.92	0.50	− 7.02 × 10^−4^	− 2.88 × 10^−3^	1.48 × 10^−3^	0.53	0.98	− 4.08 × 10^−3^	− 1.12 × 10^−2^	3.07 × 10^−3^	0.26	0.98
	*Parasutterella excrementihominis*	1.15 × 10^−3^	1.41 × 10^−4^	2.15 × 10^−3^	**0.03**	**0.17**	1.57 × 10^−3^	− 2.46 × 10^−4^	3.38 × 10^−3^	0.09	0.76	3.66 × 10^−3^	− 2.32 × 10^−3^	9.64 × 10^−3^	0.23	0.98
	*Coprococcus catus*	− 4.75 × 10^−5^	− 9.32 × 10^−4^	8.37 × 10^−4^	0.92	0.50	2.29 × 10^−4^	− 1.41 × 10^−3^	1.86 × 10^−3^	0.78	0.99	1.33 × 10^−3^	− 4.06 × 10^−3^	6.72 × 10^−3^	0.63	0.98
	*Veillonella dispar*	4.15 × 10^−4^	− 5.24 × 10^−4^	1.35 × 10^−3^	0.38	0.45	1.81 × 10^−4^	− 1.84 × 10^−3^	2.20 × 10^−3^	0.86	0.99	1.26 × 10^−3^	− 5.57 × 10^−3^	8.10 × 10^−3^	0.72	0.98
	*Clostridium disporicum*	− 5.66 × 10^−4^	− 1.59 × 10^−3^	4.54 × 10^−4^	0.28	0.41	− 2.09 × 10^−3^	− 4.11 × 10^−3^	− 6.51 × 10^−5^	0.04	0.76	− 6.38 × 10^−3^	− 1.38 × 10^−2^	1.04 × 10^−3^	0.09	0.98
	*Blautia stercoris*	3.68 × 10^−4^	− 7.02 × 10^−4^	1.44 × 10^−3^	0.50	0.45	− 6.51 × 10^−5^	− 2.10 × 10^−3^	1.97 × 10^−3^	0.95	0.99	− 7.08 × 10^−4^	− 7.20 × 10^−3^	5.78 × 10^−3^	0.83	0.98
	*Roseburia intestinalis*	1.17 × 10^−3^	− 3.66 × 10^−5^	2.37 × 10^−3^	0.06	0.19	8.77 × 10^−4^	− 1.46 × 10^−3^	3.22 × 10^−3^	0.46	0.98	1.76 × 10^−3^	− 5.99 × 10^−3^	9.51 × 10^−3^	0.65	0.98
	*Bacteroides caccae*	4.84 × 10^−4^	− 4.67 × 10^−4^	1.43 × 10^−3^	0.32	0.45	5.02 × 10^−4^	− 1.29 × 10^−3^	2.29 × 10^−3^	0.58	0.98	2.56 × 10^−3^	− 3.64 × 10^−3^	8.76 × 10^−3^	0.42	0.98
	*Lachnospira eligens*	1.47 × 10^−4^	− 8.22 × 10^−4^	1.12 × 10^−3^	0.77	0.48	− 1.91 × 10^−4^	− 2.04 × 10^−3^	1.66 × 10^−3^	0.84	0.99	− 4.62 × 10^−4^	− 6.84 × 10^−3^	5.91 × 10^−3^	0.89	0.98
	*Phascolarctobacterium faecium*	− 2.64 × 10^−4^	− 1.40 × 10^−3^	8.69 × 10^−4^	0.65	0.48	− 3.16 × 10^−4^	− 2.33 × 10^−3^	1.69 × 10^−3^	0.76	0.99	9.16 × 10^−4^	− 5.78 × 10^−3^	7.61 × 10^−3^	0.79	0.98
	*Bacteroides finegoldii*	− 4.41 × 10^−4^	− 1.60 × 10^−3^	7.20 × 10^−4^	0.45	0.45	− 1.42 × 10^−3^	− 3.56 × 10^−3^	7.08 × 10^−4^	0.19	0.82	− 4.19 × 10^−3^	− 1.12 × 10^−2^	2.80 × 10^−3^	0.24	0.98
	*Eubacterium ramulus*	7.42 × 10^−4^	2.14 × 10^−5^	1.46 × 10^−3^	**0.04**	**0.18**	7.34 × 10^−4^	− 8.04 × 10^−4^	2.27 × 10^−3^	0.35	0.95	8.16 × 10^−4^	− 4.39 × 10^−3^	6.02 × 10^−3^	0.76	0.98
	*Streptococcus salivarius*	− 9.20 × 10^−4^	− 2.02 × 10^−3^	1.80 × 10^−4^	0.10	0.21	− 7.28 × 10^−4^	− 2.94 × 10^−3^	1.49 × 10^−3^	0.52	0.98	− 1.82 × 10^−3^	− 9.48 × 10^−3^	5.85 × 10^−3^	0.64	0.98
	*Akkermansia muciniphila*	− 1.07 × 10^−5^	− 1.20 × 10^−3^	1.18 × 10^−3^	0.99	0.52	− 5.98 × 10^−4^	− 3.01 × 10^−3^	1.82 × 10^−3^	0.63	0.99	− 3.08 × 10^−3^	− 1.13 × 10^−2^	5.18 × 10^−3^	0.46	0.98
	*Alistipes shahii*	− 2.31 × 10^−4^	− 1.03 × 10^−3^	5.71 × 10^−4^	0.57	0.45	− 2.78 × 10^−4^	− 1.79 × 10^−3^	1.24 × 10^−3^	0.72	0.99	− 9.97 × 10^−4^	− 6.42 × 10^−3^	4.42 × 10^−3^	0.72	0.98
	*Bacteroides fragilis*	− 1.50 × 10^−4^	− 1.19 × 10^−3^	8.94 × 10^−4^	0.78	0.48	5.75 × 10^−4^	− 1.43 × 10^−3^	2.58 × 10^−3^	0.57	0.98	2.46 × 10^−4^	− 6.16 × 10^−3^	6.65 × 10^−3^	0.94	0.99
	*Barnesiella intestinihominis*	− 7.93 × 10^−4^	− 1.60 × 10^−3^	1.74 × 10^−5^	0.06	0.19	3.45 × 10^−4^	− 1.24 × 10^−3^	1.93 × 10^−3^	0.67	0.99	9.21 × 10^−4^	− 4.66 × 10^−3^	6.50 × 10^−3^	0.74	0.98
	*Sutterella wadsworthensis*	− 3.80 × 10^−4^	− 1.48 × 10^−3^	7.22 × 10^−4^	0.50	0.45	− 1.43 × 10^−3^	− 3.46 × 10^−3^	6.01 × 10^−4^	0.17	0.82	− 5.49 × 10^−3^	− 1.22 × 10^−2^	1.22 × 10^−3^	0.11	0.98
	*Phocaeicola massiliensis*	− 4.06 × 10^−4^	− 1.53 × 10^−3^	7.15 × 10^−4^	0.48	0.45	1.27 × 10^−4^	− 1.91 × 10^−3^	2.16 × 10^−3^	0.90	0.99	5.74 × 10^−4^	− 5.90 × 10^−3^	7.05 × 10^−3^	0.86	0.98
	*Ruminococcus callidus*	4.27 × 10^−4^	− 5.12 × 10^−4^	1.37 × 10^−3^	0.37	0.45	3.53 × 10^−4^	− 1.46 × 10^−3^	2.17 × 10^−3^	0.70	0.99	1.44 × 10^−3^	− 4.52 × 10^−3^	7.39 × 10^−3^	0.63	0.98
	*Lachnospira pectinoschiza*	1.19 × 10^−3^	2.53 × 10^−4^	2.12 × 10^−3^	**0.01**	**0.16**	9.97 × 10^−4^	− 8.46 × 10^−4^	2.84 × 10^−3^	0.29	0.89	3.29 × 10^−3^	− 3.11 × 10^−3^	9.68 × 10^−3^	0.31	0.98

CI, confidence interval; EGCG, epigallocatechin-3-gallate

^a^Beta coefficients, confidence intervals, and *p* values from the linear mixed-effects model with random intercept

^b^False discovery rate* q* values were calculated based on the observed *p* values for each taxonomical level. If a *p* value of < 0.05 and a *q* value of < 0.20 were observed, the finding was considered statistically significant and shown in bold

### Mediation effects of the gut microbiota on the association between green tea intake and blood glucose levels

**Fig. 3:**
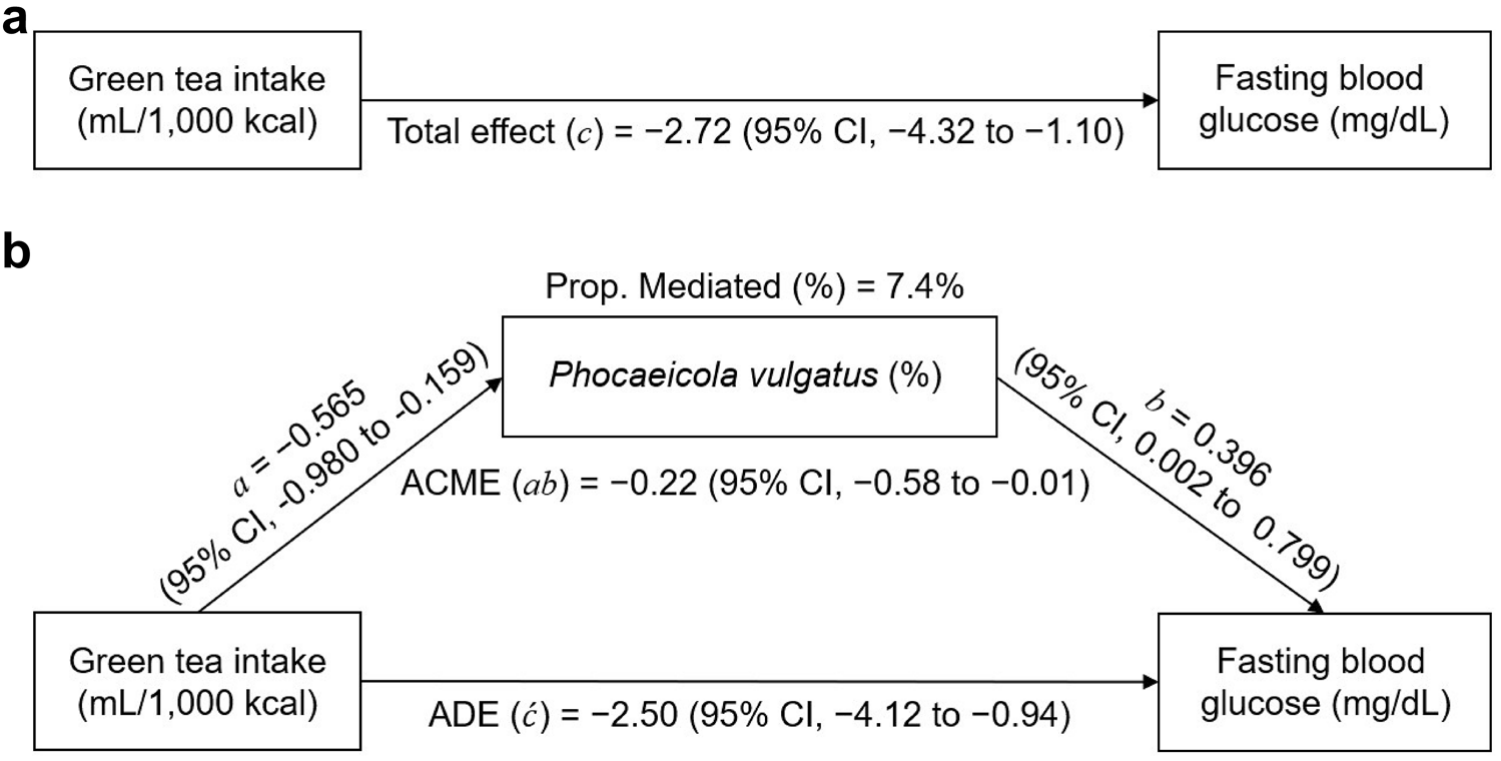
Mediation effect of *Phocaeicola vulgatus* in the association between the total effect of green tea intake and fasting blood glucose levels. **a** The total effect of green tea intake on fasting blood glucose levels (path *c*). **b** Mediation analysis was used to divide the total effect (path *c* in **a**) into the average direct effect of exposure (ADE, path ć) and the average causal mediating effect (ACME, path *ab*). Regression coefficients and its corresponding 95% confidence intervals for the association between green tea intake and the relative abundance of *P. vulgatus* (path *a*) and the association between the relative abundance of *P. vulgatus* and fasting blood glucose levels (path *b*) were estimated using the linear mixed-effects models. The relative abundance of *P. vulgatus* and fasting blood glucose levels was modeled as the continuous mediator and outcome, respectively. Green tea intake was categorized into low- and high-intake groups based on their median values (135 mL/1,000 kcal) to make a contrast in the mediation analysis (Imai et al. 2010). Abbreviations: CI, confidence interval; ACME, average causal mediation effect; ADE, average direct effect; Prop. Mediated (%), the proportion of ACME to total effect

A mediation analysis was conducted to assess the gut microbiota-mediated associations of green tea intake with fasting blood glucose and insulin levels. Based on the above-described analysis, nine species (*B. ovatus*, *E. ramulus*, *I. bartlettii*, *F. plautii*, *L. pectinoschiza*, *P. excrementihominis*, *A. hadrus*, *P. vulgatus*, and *B. luti*) were selected as candidates for the mediation analysis. Results indicated that *P. vulgatus* partially mediated the association between green tea intake and fasting blood glucose levels (Fig. 3). Specifically, the total effect and ADE of green tea intake on fasting blood glucose levels were − 2.72 (95% CI, − 4.32 to − 1.10; Fig. 3a) and − 2.50 (95% CI, − 4.12 to − 0.94; Fig. 3b), respectively. The ACME of *P. vulgatus* was − 0.22 (95% CI, − 0.58 to − 0.01, Fig. 3b), while the proportion of ACME in the total effect was 7.4% (*p* = 0.046; Fig. 3b).

## Discussion

This four-season observational study on individuals without T2DM showed that green tea intake was significantly associated with fasting blood glucose and insulin levels in the four seasons. Catechins and EGCG intakes had marginal negative associations with HbA1c levels in the first season (i.e., winter), but not in the four seasons. Green tea intake was negatively associated with the relative abundances of *P. vulgatus*, *A. hadrus*, and *B. luti*, and positively associated with the relative abundances of *B. ovatus*, *I. bartlettii*, *L. pectinoschiza*, *E. ramulus*, *F. plautii*, and *P. excrementihominis*. The mediation analysis demonstrated that *P. vulgatus* partially mediated the negative association between green tea intake and fasting blood glucose levels.

Green tea and catechins were reported to improve glucose metabolism and reduce the risk of T2DM (Iso et al. 2006; Liu et al. 2013; Rienks et al. 2018), but the effects of habitual consumption of green tea and catechins have not been examined with consideration of seasonal variations. Thus, we measured the intakes of green tea and catechins in all four seasons. We found that the amount of green tea intake varied by season, being higher in winter than in other seasons, as reported previously (Taguchi et al. 2015a, 2019); this finding indicates that seasonal variations should be considered when evaluating the effects of green tea intake in the general population. In addition, although not directly comparable, the mean green tea intake (333–443 mL/day, reflecting 2–3 cups per day) was slightly higher or equivalent to the mean value in the Japanese population (Fukushima et al. 2009; Taguchi et al. 2015b), but the catechins intake was much lower than that administered in previous intervention studies (median: 61.2–90.7 mg/day vs. 457 mg/day) (Liu et al. 2013). Our findings illustrate that even if green tea does not contain large amounts of catechins, habitual green tea intake plays a key role in maintaining the fasting blood glucose and insulin levels in all four seasons.

Previous studies generally found that the administration of green tea and catechins increased the abundance of potential beneficial microbial genera (e.g., *Bifidobacterium*, *Lactobacillus*) (Tzounis et al. 2008; Zhang et al. 2013; Sun et al. 2018) or decreased the abundance of specific pathogenic bacteria such as *Clostridium* spp. (Sakanaka et al. 2000; Cui et al. 2012; Zhang et al. 2013). In the present study, green tea intake was associated with specific gut microbiota at the species level rather than at the phylum and genus levels; this finding indicates that even in bacteria belonging to the same phylum and genus, green tea intake may differently affect the relative abundances of various microbial species, such as *F. plautii* and *P. excrementihominis*. *F. plautii* is a major flavonoid degrader in the human gut microbiota (Goris et al. 2021); it has the ability to metabolize quercetin, a flavonoid abundantly present in green tea, and produce butyrate (Carlier et al. 2010). The abundance of *F. plautii* was reported to be enriched in mice after the oral administration of green tea (Mikami et al. 2021). *P. excrementihominis* is likely to rely on amino acids to support its metabolic activities and physiological functions (Ju et al. 2019). Given that green tea contains large amounts of free amino acids (Zhou et al. 2022), it would promote the growth of *P. excrementihominis*, but further studies are needed to clarify this association. Taken together, habitual green tea intake, but not intake of catechins or EGCG alone, may contribute to the modulation of specific gut microbiota species.

Although many animal studies have indicated that the antidiabetic effects of green tea and catechins are partially mediated by the gut microbiota (Henning et al. 2018; Chen et al. 2019; Li et al. 2020; Park et al. 2020; Zhang et al. 2020; Wu et al. 2021), little is known about which specific bacteria play important roles as mediators, especially in humans. This four-season observational study demonstrated that *P. vulgatus* is the potential mediator of the association between green tea intake and fasting blood glucose levels in individuals without T2DM in all four seasons. *P. vulgatus* is a dominant species in the human gut microbiota, where it is widely considered responsible for the development of inflammation (Cai et al. 2020). Mechanistic studies employing a gnotobiotic model and a cell culture system demonstrated that *P. vulgatus* produces mucin-degrading enzymes that can profoundly damage the mucosal barrier function (Ruseler-van Embden et al. 1989; Hoskins et al. 1992). An imbalance in the mucosal barrier function can be a trigger for low-grade inflammation and metabolic disorders, including T2DM (Cani et al. 2008). A previous cohort study on 277 non-diabetic Danish individuals found that *P. vulgatus* was the primary species driving the association between biosynthesis of branched-chain amino acids and insulin resistance (Pedersen et al. 2016); meanwhile, an RCT involving obese women found a correlation between *P. vulgatus* and poor glycemic control (Dewulf et al. 2013). A clinical study showed that *P. vulgatus* can provide a gut microbial signature associated with T2DM development (Leite et al. 2017). Furthermore, a recent animal study demonstrated that green tea intake decreased the relative abundance of *P. vulgatus* and improved the fasting glucose levels (Ma et al. 2020). The findings of the present study indicate that habitual green tea intake improves glucose metabolism by suppressing the abundance of *P. vulgatus* that is associated with elevated blood glucose levels in individuals without T2DM.

The present study has several limitations. First, because all participants were recruited from Shizuoka, Japan, the generalizability of the findings may be limited to the entire Japanese population. Second, there were 1.2%–20.5% missing data in the variables due to the repeated collection of data from the same participants in all four seasons. Of note, high levels of motivation were required to collect the 3-day WDRs and tea samples, which may have resulted in poor response rates (Willett 2012). To address the missing data and obtain valid estimates, multiple imputations were performed to compensate for missing values under the MAR assumption, and the plausibility of the MAR assumption was confirmed using sensitivity analyses (Rubin 1987; van Buuren and Groothuis-Oudshoorn 2011). Finally, the 16S rRNA gene sequencing technique is highly useful for bacterial classification, but it has low phylogenetic power at the species level compared with that at the phylum and genus levels (Mignard and Flandrois 2006; Janda and Abbott 2007).

This four-season observational study of individuals without T2DM suggested that daily intake of green tea, but not intake of catechins or EGCG alone, may improve the glucose metabolism and change the relative abundances of certain microbial species in all four seasons. Among the bacteria associated with green tea intake, *P. vulgatus* was identified as a potential mediator in the negative association between green tea intake and fasting blood glucose levels. Green tea may improve the glucose metabolism by suppressing the abundance of *P. vulgatus* that is associated with elevated blood glucose levels in individuals without T2DM.

## Supplementary Information

Below is the link to the electronic supplementary material.

Supplementary file1 (DOCX 38 KB)

## Data Availability

The datasets generated or analyzed during the current study are not publicly available due to ethical restrictions, but are available from the corresponding author upon reasonable request.
