# Supplementary material for: Gut microbiota-mediated associations of green tea and catechin intakes with glucose metabolism in individuals without type 2 diabetes mellitus: a four-season observational study with mediation analysis
Source: Arch Microbiol. 2023 Apr 14;205(5):191. doi: 10.1007/s00203-023-03522-y (PMC10104920; doi:10.1007/s00203-023-03522-y)
Supplement: Supplementary file 1 — Supplementary file1 (DOCX 38 KB) [file 203_2023_3522_MOESM1_ESM.docx]

**Supplementary Information for Archives of Microbiology**

**Gut microbiota-mediated associations of green tea and catechin intakes with glucose metabolism in individuals without type 2 diabetes mellitus: A four-season observational study with mediation analysis**

Aoi Ito,^1^ Yuji Matsui,^2^ Masao Takeshita,^2^ Mitsuhiro Katashima,^2^ Chiho Goto,^3^ Kiyonori Kuriki^1,4^

^1^Laboratory of Public Health, Graduate School of Integrated Pharmaceutical and Nutritional Sciences, University of Shizuoka, Shizuoka, Japan

^2^R&D - Health & Wellness Products Research, Kao Corporation, Tokyo, Japan

^3^Department of Health and Nutrition, Nagoya Bunri University, Aichi, Japan

^4^Laboratory of Public Health, School of Food and Nutritional Sciences, University of Shizuoka, Shizuoka, Japan

**Corresponding author:** Kiyonori Kuriki, PhD

Laboratory of Public Health, Graduate School of Integrated Pharmaceutical and Nutritional Sciences, University of Shizuoka, 52-1 Yada, Suruga-ku, Shizuoka, Japan.

Tel: +81-54-264-5563

Email: [kuriki@u-shizuoka-ken.ac.jp](mailto:kuriki@u-shizuoka-ken.ac.jp)

**Table S1** Sensitivity analysis with δ-adjustment of the associations between green tea and catechin intakes and biomarkers of glucose metabolism

| Delta value | Outcome | Exposure | β^a^ | 95% CI^a^ | | *p*^b^ |
| --- | --- | --- | --- | --- | --- | --- |
|  |  |  |  | 2.5% | 97.5% |  |
| δ = −200 | Fasting blood glucose | Green tea | −5.09×10^−3^ | −1.00×10^−2^ | −1.54×10^−4^ | **0.04** |
|  |  | Catechins | −1.30×10^−3^ | −9.93×10^−3^ | 7.33×10^−3^ | 0.77 |
|  |  | EGCG | 5.95×10^−3^ | −2.64×10^−2^ | 3.83×10^−2^ | 0.72 |
|  | HbA1c | Green tea | 8.17×10^−5^ | −8.38×10^−5^ | 2.47×10^−4^ | 0.33 |
|  |  | Catechins | 7.10×10^−5^ | −1.96×10^−4^ | 3.38×10^−4^ | 0.60 |
|  |  | EGCG | 1.89×10^−4^ | −7.04×10^−4^ | 1.08×10^−3^ | 0.68 |
|  | Insulin | Green tea | −4.64×10^−4^ | −8.07×10^−4^ | −1.21×10^−4^ | **0.01** |
|  |  | Catechins | −4.78×10^−4^ | −1.19×10^−3^ | 2.37×10^−4^ | 0.19 |
|  |  | EGCG | −4.32×10^−4^ | −3.00×10^−3^ | 2.13×10^−3^ | 0.74 |
|  | HOMA-IR | Green tea | −1.12×10^−4^ | −5.12×10^−4^ | 2.89×10^−4^ | 0.58 |
|  |  | Catechins | 7.00×10^−6^ | −7.54×10^−4^ | 7.68×10^−4^ | 0.99 |
|  |  | EGCG | 9.35×10^−4^ | −1.65×10^−3^ | 3.52×10^−3^ | 0.48 |
| δ = −100 | Fasting blood glucose | Green tea | −5.19×10^−3^ | −9.99×10^−3^ | −3.77×10^−4^ | **0.03** |
|  |  | Catechins | −1.19×10^−3^ | −1.02×10^−2^ | 7.78×10^−3^ | 0.79 |
|  |  | EGCG | 6.84×10^−3^ | −2.35×10^−2^ | 3.71×10^−2^ | 0.66 |
|  | HbA1c | Green tea | 1.02×10^−4^ | −6.13×10^−5^ | 2.65×10^−4^ | 0.22 |
|  |  | Catechins | 8.02×10^−5^ | −1.78×10^−4^ | 3.38×10^−4^ | 0.54 |
|  |  | EGCG | 2.34×10^−4^ | −6.03×10^−4^ | 1.07×10^−3^ | 0.58 |
|  | Insulin | Green tea | −4.47×10^−4^ | −7.98×10^−4^ | −9.67×10^−5^ | **0.01** |
|  |  | Catechins | −4.51×10^−4^ | −1.13×10^−3^ | 2.27×10^−4^ | 0.19 |
|  |  | EGCG | −4.53×10^−4^ | ^−2^.82×10^−3^ | 1.92×10^−3^ | 0.71 |
|  | HOMA-IR | Green tea | −1.20×10^−4^ | −5.19×10^−4^ | 2.79×10^−4^ | 0.55 |
|  |  | Catechins | −7.20×10^−7^ | −8.06×10^−4^ | 8.05×10^−4^ | 1.00 |
|  |  | EGCG | 9.45×10^−4^ | −1.63×10^−3^ | 3.52×10^−3^ | 0.47 |
| δ = 0 (MCAR) | Fasting blood glucose | Green tea | −5.27×10^−3^ | −9.99×10^−3^ | −5.41×10^−4^ | **0.03** |
|  |  | Catechins | −1.47×10^−3^ | −1.05×10^−2^ | 7.58×10^−3^ | 0.75 |
|  |  | EGCG | 4.67×10^−3^ | −2.44×10^−2^ | 3.37×10^−2^ | 0.75 |
|  | HbA1c | Green tea | 9.25×10^−5^ | −6.82×10^−5^ | 2.53×10^−4^ | 0.26 |
|  |  | Catechins | 8.96×10^−5^ | −1.71×10^−4^ | 3.50×10^−4^ | 0.50 |
|  |  | EGCG | 1.56×10^−4^ | −6.76×10^−4^ | 9.89×10^−4^ | 0.71 |
|  | Insulin | Green tea | −4.67×10^−4^ | −8.17×10^−4^ | −1.16×10^−4^ | **0.01** |
|  |  | Catechins | −4.96×10^−4^ | −1.20×10^−3^ | 2.12×10^−4^ | 0.17 |
|  |  | EGCG | −6.45×10^−4^ | −3.13×10^−3^ | 1.84×10^−3^ | 0.61 |
|  | HOMA-IR | Green tea | −1.51×10^−4^ | −5.57×10^−4^ | 2.55×10^−4^ | 0.46 |
|  |  | Catechins | −5.79×10^−5^ | −8.16×10^−4^ | 7.00×10^−4^ | 0.88 |
|  |  | EGCG | 1.01×10^−3^ | −1.35×10^−3^ | 3.37×10^−3^ | 0.40 |
| δ = 100 | Fasting blood glucose | Green tea | −5.01×10^−3^ | −9.93×10^−3^ | −8.56×10^−5^ | **0.05** |
|  |  | Catechins | −6.06×10^−4^ | −9.86×10^−3^ | 8.65×10^−3^ | 0.90 |
|  |  | EGCG | 5.52×10^−3^ | −2.65×10^−2^ | 3.76×10^−2^ | 0.73 |
|  | HbA1c | Green tea | 8.81×10^−5^ | −6.95×10^−5^ | 2.46×10^−4^ | 0.27 |
|  |  | Catechins | 8.10×10^−5^ | −2.04×10^−4^ | 3.66×10^−4^ | 0.57 |
|  |  | EGCG | 1.26×10^−4^ | −7.29×10^−4^ | 9.81×10^−4^ | 0.77 |
|  | Insulin | Green tea | −4.55×10^−4^ | −8.00×10^−4^ | −1.10×10^−4^ | **0.01** |
|  |  | Catechins | −4.43×10^−4^ | −1.17×10^−3^ | 2.86×10^−4^ | 0.23 |
|  |  | EGCG | −6.21×10^−4^ | −3.10×10^−3^ | 1.86×10^−3^ | 0.62 |
|  | HOMA-IR | Green tea | −1.34×10^−4^ | −5.55×10^−4^ | 2.86×10^−4^ | 0.53 |
|  |  | Catechins | −5.08×10^−5^ | −8.61×10^−4^ | 7.60×10^−4^ | 0.90 |
|  |  | EGCG | 7.89×10^−4^ | −1.66×10^−3^ | 3.24×10^−3^ | 0.53 |
| δ = 200 | Fasting blood glucose | Green tea | −5.12×10^−3^ | −9.96×10^−3^ | −2.76×10^−4^ | **0.04** |
|  |  | Catechins | −1.39×10^−3^ | −1.08×10^−2^ | 7.98×10^−3^ | 0.77 |
|  |  | EGCG | 3.02×10^−3^ | −2.50×10^−2^ | 3.11×10^−2^ | 0.83 |
|  | HbA1c | Green tea | 8.82×10^−5^ | −6.83×10^−5^ | 2.45×10^−4^ | 0.27 |
|  |  | Catechins | 7.35×10^−5^ | −1.87×10^−4^ | 3.35×10^−4^ | 0.58 |
|  |  | EGCG | 1.32×10^−4^ | −7.18×10^−4^ | 9.83×10^−4^ | 0.76 |
|  | Insulin | Green tea | −4.64×10^−4^ | −8.22×10^−4^ | −1.07×10^−4^ | **0.01** |
|  |  | Catechins | −4.56×10^−4^ | −1.20×10^−3^ | 2.88×10^−4^ | 0.23 |
|  |  | EGCG | −8.15×10^−4^ | −3.17×10^−3^ | 1.54×10^−3^ | 0.49 |
|  | HOMA-IR | Green tea | −1.15×10^−4^ | −5.20×10^−4^ | 2.89×10^−4^ | 0.57 |
|  |  | Catechins | −4.17×10^−5^ | −7.86×10^−4^ | 7.02×10^−4^ | 0.91 |
|  |  | EGCG | 1.03×10^−3^ | −1.44×10^−3^ | 3.49×10^−3^ | 0.41 |

^a^The beta coefficients, confidence intervals (CI), and *p*-values were estimated from the linear mixed-effects model with random intercept.

^b^*P*-values shown in bold are statistically significant (*p*<0.05)

Abbreviations: HbA1c, glycated hemoglobin A1c; HOMA-IR, homeostatic model assessment index of insulin resistance; EGCG, epigallocatechin-3-gallate; MCAR, missing completely at random
